# Supplementary material for: A conserved mechanism for regulating replisome disassembly in eukaryotes
Source: Nature. 2021 Oct 26;600(7890):743–7. doi: 10.1038/s41586-021-04145-3 (PMC8695382; doi:10.1038/s41586-021-04145-3)
Supplement: Supplementary file 1 — This file contains Supplementary Methods; Supplementary Tables 1 and 2; Supplementary Figures 1 and 2; and Supplementary References. [file 41586_2021_4145_MOESM1_ESM.pdf]

nature portfolio

https://doi.org/10.1038/s41586-021-04145-3

Supplementary information

A conserved mechanism for regulating replisome disassembly in eukaryotes

In the format provided by the authors and unedited

nature portfolio

https://doi.org/10.1038/s41586-021-04145-3

Supplementary information

A conserved mechanism for regulating replisome disassembly in eukaryotes

In the format provided by the authors and unedited

METHODS

Purification of yeast proteins

Cdc34, Cdc45, Cdc6, Cdc9, Cdt1·Mcm2-7, CMG, Tof1-Csm3, Ctf4, DDK, Dpb11, Fen1, GINS, Mcm10, ORC, PCNA, Pif1, Pol α - primase, Pol δ, Pol ε, Pol ε^{exo-}, RFC, RPA, S-CDK, SCF^{Dia2}, Sld2, Sld3-7, Top1 and Uba1 were purified as previously described[1-7]. Ubiquitin was kindly provided by Dr. Axel Knebel (MRC PPU, Dundee, U.K.).

Mrc1 purification

Mrc1 was purified as previously described[7] except substituting 150 mM NaOAc for NaCl in the final gel filtration step for protein stocks used in the cryo-EM sample preparation of complexes assembled on dsDNA.

Cdc34-Ub purification

To prepare Cdc34-Ub, a 5 ml reaction was assembled containing 7.5 μM Cdc34 (harbouring the C95K mutation), 1 μM Uba1 and 50 μM HIS_6-ubiquitin in 50 mM Tris-Cl (pH 10), 5 mM $Mg(OAc)_2$, 2 mM ATP and 1 mM TCEP at 30°C for 16 h. Subsequently, this sample was loaded onto a 120 ml Superdex 200 column in 25 mM Hepes-KOH (pH 7.6), 10% glycerol, 500 mM NaCl, 0.5 mM TCEP. Peak fractions containing Cdc34-HIS_6-Ub were pooled, imidazole was added to 30 mM, and Cdc34-HIS_6-Ub was purified by incubation with 0.5 ml Ni-NTA beads for 30 min at room temperature. Cdc34-HIS_6-Ub was eluted in 25 mM Hepes-KOH (pH 7.6), 10% glycerol, 500 mM NaCl, 0.5 mM TCEP, 400 mM imidazole and then dialysed vs. 25 mM Hepes-KOH (pH 7.6), 10% glycerol, 150 mM NaCl, 0.5 mM TCEP at 4°C overnight. The dialysed sample was recovered, concentrated to 1.7 μM, aliquoted and snap froze.

Expression of human proteins in insect cells

Bacmids were prepared following transformation of EMBacY *E. coli* with vector constructs either expressing individual proteins or protein complexes (see supplementary table 2 for vector details). Baculoviruses were generated by transfecting Sf9 cells with purified bacmids using FuGENE® HD (Promega) before subsequent amplification. For protein expression, Hi5

cells (1 x 10^6 cells / ml) were infected with the required baculovirus and growth continued for 72 h before harvest by centrifugation.

Purification of human proteins

CMG, TIMELESS-TIPIN, CLASPIN, AND-1 and Pol ε were purified as previously described[8]. CMG was also purified using a modified version of the method in [8]. Here, cells lysis, anti-FLAG immunoprecipitation and Streptactin affinity chromatography were performed as described previously[8]. The flow through from the Streptactin column was applied to a Mono Q 5/50 GL column (GE Healthcare) equilibrated in 25 mM Tris-HCl pH 7.2, 10% glycerol, 0.005% TWEEN 20, 0.5 mM TCEP, 150 mM KCl and bound proteins were eluted with a 20 column volume gradient to 1 M KCl. Peak fractions from the Streptactin and MonoQ columns were pooled, concentrated to ~ 500 μl (Amicon Ultra, Ultracel - 30K) and applied to a Superdex 200 Increase 10/300 column (GE Healthcare) equilibrated in 25 mM Hepes-KOH pH 7.6, 200 mM potassium acetate, 10% glycerol, 0.5 mM EDTA, 0.005% Tween 20, 0.5 mM TCEP. Peak fractions were pooled and applied to a MonoQ PC 1.6/5 (GE healthcare). The monoQ and subsequent purification steps were performed as previously described[8].

$CUL2^{LRR1}$ purification

Cells from a 2L culture were resuspended in 40 ml lysis buffer (40 mM Tris-Cl (pH 7.2), 200 mM NaCl, 10% glycerol, 1 mM EDTA, 0.005% NP40, 1 mM DTT) + protease inhibitors (1 tablet cOmplete, EDTA- free (Roche) per 25 ml buffer). Cells were lysed by Dounce homogenisation and cell debris was cleared by centrifugation (235,000 g, 4°C, 45 min). To the soluble lysate, 1.7 ml FLAG-M2 resin was added and the sample was incubated on a rotating wheel for 90 min at 4°C. The resin was collected in a 20 ml gravity flow column (Biorad) and washed three times with 20 ml lysis buffer followed by 10 ml lysis buffer + 5 mM $MgCl_2$ + 0.5 mM ATP. During the ATP wash the column flow was stopped for 10 min before the column was washed twice more with 20 ml lysis buffer. Bound proteins were eluted in 2 ml lysis buffer + 0.2 mg/ml FLAG peptide followed by a second 4 ml elution in lysis buffer + 0.1 mg/ml FLAG peptide. The eluates were pooled, concentrated to 600 μL (amicon ultra 30 kDa cutoff) and applied to a Superdex 200 Increase 10/300 column equilibrated in 25 mM Hepes KOH (pH 7.6), 300 mM KOAc, 10% glycerol, 1 mM EDTA, 0.005% Tween 20, 1 mM DTT. Peak fractions were pooled, frozen in liquid nitrogen and stored at -80°C.

Cryo-EM sample preparation

***S. cerevisiae* replisome:SCF^{Dia2} complexes on dsDNA**

The DNA substrate was annealed by mixing equal volumes of the leading strand template (5'-TAGAGTAGGAAmGmTmGmAmTmGmGmTmAmAmGmTmGmAmTmTmAmGmAmGAATTGG AGAGTGTG$(T)_{34}$T*T*T*T*T*T; *=phosphorothioate linkage, m=methylphosphonate linkage) and the lagging strand template (5'-ACACACTCTCCAATTCTCTAATCACTTACCATCACT TCCTACTCTA), each at 100 μM in 10 mM Tris-HCl (pH 8.0), 1 mM EDTA, before allowing to cool gradually from 75°C to room temperature.

CMG (1.1 μM) was incubated with 1.5-fold molar excess DNA in reconstitution buffer (25 mM HEPES-NaOH (pH 7.6), 100 mM NaOAc, 0.5 mM TCEP, 7.5 mM $Mg(OAc)_2$, 0.5 mM ATP) in a 30 μL reaction volume, and incubated on ice for 30 min. To this was added a mixture of Tof1-Csm3, Ctf4, Mrc1, Pol ε^{exo-}, Cdc34-Ub and SCF^{Dia2} in reconstitution buffer (containing an additional ~30 mM NaOAc contributed by Mrc1 storage buffer (58.9 μL) and 40 mM KOAc contributed by SCF^{Dia2} storage buffer (31.3 μL); contribution of other stocks negligible), giving a final reaction volume of 300 μL and resulting in a final concentration of 110 nM CMG with all other factors in 1.5-fold molar excess (except 2-fold molar excess Mrc1). The reaction was incubated on ice for a further 40 min before loading 100 μL onto each of two GraFix gradients (plus one gradient with crosslinking agents omitted for assessing the position of peak fractions) prepared as described previously[7]. Gradient sedimentation and subsequent buffer exchange were performed as described[7], except pooling peak fractions across both crosslinking gradients (total volume ~370 μL). Sample was concentrated to ∼24 μL and immediately used for cryo-EM grid preparation as previously described[7].

***S. cerevisiae* replisome:SCF^{Dia2} complexes in the absence of DNA**

CMG (final concentration 100 nM) was mixed with 1.5-fold molar excess Tof1-Csm3, Ctf4, Pol ε^{exo-}, Cdc34-Ub and SCF^{Dia2}, plus 2-fold molar excess Mrc1, in 25 mM HEPES-NaOH (pH 7.6), ~40 mM NaCl*, ~50 mM KOAc*, 0.5 mM TCEP, 2.75 mM $Mg(OAc)_2$, 0.5 mM AMP-PNP with a final reaction volume of 230 μL. The reaction was incubated on ice for 60 min before loading equally across two GraFix gradients, prepared as described[7].

Gradient sedimentation and subsequent buffer exchange were performed as described[7], except pooling peak fractions across both crosslinking gradients (total volume ~550 µL). Sample was concentrated to ~27 µL and immediately used for cryo-EM grid preparation as previously described[7].

*Salt contributed by storage buffers of protein stocks (30.5 µL SCF^{Dia2}, 63 µL Mrc1); contribution of other stocks negligible.

H. sapiens replisome:CUL2^{LRR1} complexes on 3'-flap DNA

The DNA substrate was annealed by mixing equal volumes of the leading strand template (5'-(Cy3)TAGAGTAGGAAGTGA(Biotinylated-dT)GGTAAGTGATTAGAGAATTGGAGAGTGTG$(T)_{34}$ T*T*T*T*T*T; *=phosphorothioate linkage) and the lagging strand template (5'-ACACACTCTCCAATTCTCTAATCACTTACCA(Biotinylated-dT)CACTTCCTACTCTA), each at 53 µM in 25 mM HEPES-NaOH (pH 7.5), 150 mM NaOAc, 0.5 mM TCEP, 2 mM $Mg(OAc)_2$, before allowing to cool gradually from 75°C to room temperature.

CMG (1.7 µM) was incubated with 1.5-fold molar excess DNA in reconstitution buffer (25 mM HEPES-NaOH (pH 7.6), 150 mM NaOAc, 0.5 mM TCEP, 10 mM $Mg(OAc)_2$, 0.5 mM AMP-PNP) in a 85 µL reaction volume, and incubated on ice for 30 min. To this, a mixture of TIMELESS-TIPIN, AND-1, CLASPIN, Pol ε and $CUL2^{LRR1}$ was added in reconstitution buffer, giving a final reaction volume of 240 µL. This resulted in a final concentration of 230 nM CMG with all other factors in 1.5-fold molar excess. The reaction was incubated on ice for a further 30 min before loading 106 µL onto each of two GraFix gradients. The remaining 28 µL was diluted in reconstitution buffer to 106 µL and loaded onto a single gradient in the absence of crosslinking agents to determine the position of peak fractions. Gradient preparation, sedimentation and subsequent buffer exchange were performed as described[7]. Two peak fractions from each crosslinking gradient (total volume ~370 µL) were pooled and concentrated to ~24 µL and the sample was immediately used for cryo-EM grid preparation as previously described[7].

Cryo-EM data collection

S. cerevisiae replisome:SCF^{Dia2} complexes on dsDNA

A total of 13,385 raw micrographs were acquired in a single dataset using a 300 keV Titan Krios microscope (FEI) equipped with a K3 direct electron detector (Gatan) operated in electron counting mode using the EPU automated acquisition software (ThermoFisher) with "Faster Acquisition" mode (AFIS) enabled. A slit width of 20 eV was used for the BioQuantum energy filter. Data were collected in super-resolution mode with an effective pixel spacing of 0.53 Å/pixel (nominal magnification of 81,000 X), using a defocus range of -0.4 to -2.2 μm and dose-fractionating into 38 fractions per micrograph. An exposure time of 4 s achieved a dose of 38.8 $e^-/Å^2$ per micrograph.

S. cerevisiae replisome:SCF^{Dia2} complexes in the absence of DNA

A total of 3,096 raw micrographs were acquired in a single dataset on a 300 keV Titan Krios TEM (FEI), equipped with a Falcon III direct electron detector operated in electron counting mode, using the EPU automated acquisition software (ThermoFisher) with "Faster Acquisition" mode (AFIS) and on-the-fly motion correction enabled. Data were collected at a pixel spacing of 0.87 Å/pixel (nominal magnification of 96,000 X), using a defocus range of -0.5 to -2.5 μm and dose-fractionating into 55 fractions per micrograph. An exposure time of 25.5 s achieved a dose of 32.5 $e^-/Å^2$ per micrograph.

H. sapiens replisome:CUL2^{LRR1} complexes on 3'-flap DNA

A total of 16,721 raw micrographs were acquired in a single dataset using a 300 keV Titan Krios microscope (FEI) equipped with a K3 direct electron detector (Gatan) operated in super-resolution mode using the EPU automated acquisition software (ThermoFisher) with "Faster Acquisition" mode (AFIS) enabled. A slit width of 20 eV was used for the BioQuantum energy filter. The effective pixel spacing of the data was 0.536 Å/pixel (nominal magnification of 81,000 X), using a defocus range of -0.8 to -2.8 μm and dose-fractionating into 38 fractions per micrograph. An exposure time of 4 s achieved a dose of 38.3 $e^-/Å^2$ per micrograph.

Cryo-EM data processing

S. cerevisiae replisome:SCF^{Dia2} complexes on dsDNA

Data processing used RELION-3.0/3.1[9] as outlined in Extended Data Fig. 2. The dataset was divided into three roughly equal parts (Parts A-C) which were initially processed independently; for additional details regarding particle numbers associated with each of Parts A-C, refer to Extended Data Fig. 2. The 38-fraction movies were aligned and dose-weighted (1.02 e^{-}/Å^2/fraction, 5 x 5 patches, 150 Å^2 B-factor) using RELION's implementation of a MotionCor2-like program[10] during which data were binned 2-fold to give a binned pixel spacing of 1.06 Å/pixel for use in downstream processing. CTF parameters were estimated using CTFFIND-4.1[11]. After excluding poor quality micrographs, particles were picked from the remaining 12,730 micrographs using Gautomatch v0.56[12] leading to extraction of ~2,160,000 particles using a box size of 399 Å. During extraction, data were down-sampled to a pixel size of 4.24 Å/pixel prior to three rounds of 2D classification. The best 2D classes (totalling ~1,720,000 particles) were selected and submitted for 3D classification (regularisation parameter, T = 4). At this stage, different combinations of 3D classes were combined for downstream processes depending on the specific processing goals, as discussed below. For all instances of signal subtraction, particles were reverted to the original (non-subtracted) state following 3D subclassification prior to 3D-refinement.

To isolate complexes which had translocated onto dsDNA, the best aligned 3D classes were combined irrespective of CMG-associated factor occupancy for each Part (A-C) of the dataset, giving a total of ~1,450,000 particles (orange path, Extended Data Fig. 2). Particles were subsequently re-extracted without down-sampling before performing signal subtraction focusing on the MCM channel and MCM PS1/H2I DNA-binding loops (recentring on mask) followed by 3D-subclassification (without alignment, T=100). In each of Parts A-C, this approach identified two classes with clear dsDNA engaged by the MCM C-tier, each containing ~230,000 particles, plus several additional classes featuring particles bound to ssDNA. The classes bound to ssDNA were excluded and the remaining classes combined across Parts A-C prior to iterative CTF refinement (beamtilt and trefoil correction, anisotropic magnification correction, and per-particle defocus and astigmatism CTF correction) and dataset-trained Bayesian polishing. A further round of signal subtraction focusing on the MCM channel (recentring on mask, re-boxing in 159 Å) and 3D-subclassification (without alignment, T=100)

was able to exclude additional ssDNA-bound particles, again leaving two classes bound to dsDNA. The first of these dsDNA-bound classes (~100,000 particles) represented conformation I. The second dsDNA-bound class appeared heterogeneous and was thus subjected to a final round of signal subtraction (recentring on mask, re-boxing in 159 Å) and 3D-subclassification (without alignment, T=20) this time focusing on Mcm3/Mcm7, allowing isolation of a second MCM C-tier conformation, conformation II (~65,000 particles).

Having separated conformations I and II, each population was submitted for multi-body refinement[13], defining the MCM N- and C-tiers as individual bodies. The resulting maps were submitted for map sharpening and RELION local resolution estimation[14], yielding sharpened maps with resolutions from 3.3 - 3.6 Å (refer to Extended Data Fig. 2). The MCM N-tier sharpened maps (from map sharpening) and MCM C-tier filtered maps (from RELION local resolution estimation[14]) were used for model building of these regions.

For the remaining regions of the complex, data processing focused on particles from the initial 3D classification (pixel spacing 4.24 Å/pixel) with reasonable SCF^{Dia2} occupancy (magenta path, Extended Data Fig. 2). This approach was taken after identifying no appreciable differences in SCF^{Dia2} association between the different dsDNA-bound conformations and ssDNA-bound complexes identified above. For those classes with weaker SCF^{Dia2} occupancy, signal subtraction focusing on SCF^{Dia2}/$Mcm3^{N\text{-}tier}$ (recentring on mask) and 3D subclassification (without alignment, T=4) improved the SCF^{Dia2} occupancy prior to further downstream processing. At this stage, a subset of ~168,000 particles with the best SCF^{Dia2} occupancy from Part C of the dataset were submitted for multi-body refinement defining Cdc53-Hrt1 as a single body, yielding an 8.5 Å reconstruction following map sharpening (B-factor of -50 $Å^2$).

After combining all particles with good SCF^{Dia2} occupancy across Parts A-C of the dataset (~903,000 particles), the subclassification focusing on SCF^{Dia2}/$Mcm3^{N\text{-}tier}$ was repeated to exclude additional particles lacking SCF^{Dia2}. The remaining ~783,000 particles were re-extracted without down-sampling and subjected to iterative CTF refinement and Bayesian polishing as described above, rescaling to a 1.33 Å pixel spacing during polishing. CTF-refined/polished particles were submitted for a further signal subtraction which focused on the Dia2 region (recentring on mask, re-boxing in 200 Å) and 3D subclassification (without

alignment, T=100), revealing flexibility in $Dia2^{LRR}$. The class with the best Dia2-Skp1 reconstruction (~56,000 particles) was submitted for multi-body refinement[13], defining Dia2-Skp1 as a single body, yielding a 4.0 Å reconstruction following map sharpening (B-factor of -40 $Å^2$).

Otherwise, to derive maps with good density for the additional replisome factors, all classes featuring good Dia2-Skp1 density from the above Dia2-focused 3D-subclassification were combined (~369,000 particles). These particles were used as input for multi-body refinement[13] yielding sharpened maps encompassing Cdc45-GINS-Ctf4 (3.2 Å) or Pol ε^{exo-} (3.5 Å) - B-factor of -50 $Å^2$. The same particles were separately used as input for signal subtraction (recentring on mask, re-boxing in 213 Å) and 3D-subclassification (without alignment, T=100) focusing on Tof1-Csm3, producing a 3.6 Å sharpened map (B-factor of -35 $Å^2$) with the best Tof1-Csm3/parental dsDNA density.

The sharpened maps described above, or the equivalent filtered maps generated during local resolution estimation by RELION[14], were used in subsequent model building; maps were used either individually or after combination using Phenix combine_focused_maps[15] [https://www.phenix-online.org/documentation/reference/combine_focused_maps.html].

S. cerevisiae replisome:SCF^{Dia2} complexes in the absence of DNA

Data processing used RELION-3.0[9]. The 55-fraction movies were aligned and dose-weighted (1.18 $e^-/Å^2$/group, 2 fractions per group, 5 x 5 patches, 300 $Å^2$ B-factor) using MotionCor2[10] and CTF parameters estimated using Gctf[16]. Particles were picked using Gautomatch v0.53[12] leading to extraction of ~203,000 particles (397 Å box size) with pixel spacing down-sampled by a factor of 4. To achieve good alignment during 2D classification, it was necessary to remove black pixels over 2.5-times the image standard deviation, replacing them with values from a Gaussian distribution.

Following 2D classification, the best ~86,000 well-aligned particles were re-extracted (here down-sampling by a factor of 2) without removing black pixels and submitted for 3D classification (regularisation parameter, T = 4). One well-aligned 3D class was observed (~43,000 particles) and subsequently sub-classified without further alignment. Here, the best

classes featuring good SCF^{Dia2} occupancy were combined (~42,000 particles), re-extracted without down-sampling and 3D-refined, yielding a final reconstruction at 4.6 Å resolution following map sharpening (B-factor of -135 $Å^2$). These refined particles were submitted for multi-body refinement[13], defining the MCM N-tier, Cdc45, GINS, Ctf4 and SCF^{Dia2} regions as a single body, yielding a 3.9 Å reconstruction following map sharpening (B-factor of -20 $Å^2$), displayed in Fig. 1f. The local resolution map was produced for this reconstruction using RELION[14].

***H. sapiens* replisome:$CUL2^{LRR1}$ complexes on 3'-flap DNA**

Data processing used RELION-3.1[9] as illustrated by the schematic in Extended Data Fig. 8. The 38-fraction movies were aligned and dose-weighted (1.00773 $e^-/Å^2$/fraction, 5 x 5 patches, 150 $Å^2$ B-factor) using RELION's implementation of a MotionCor2-like program[10] during which data were binned 2-fold to give an effective pixel spacing of 1.072 Å/pixel. CTF parameters were estimated using CTFFIND-4.1[11]. Particles were picked using Gautomatch v0.56[12] leading to extraction of ~2,412,000 particles using a box size of 450.2 Å. During extraction, data were down-sampled to a pixel size of 4.29 Å/pixel.

Two rounds of 3D classification (regularisation parameter, T = 4) were carried out using a previously obtained map of the core human replisome as a reference. Classes were selected based upon both the presence of high-resolution features in CMG and density corresponding to LRR1, giving a total of ~1,000,000 particles. In order to enrich for replisomes, bound by $CUL2^{LRR1}$, signal subtraction was carried out focussing on the interface between LRR1 and the MCM3 N-tier, followed by 3D classification without alignment (pink pathway Extended Data Fig. 8). For all instances of signal subtraction, particles were reverted to the original (non-subtracted) state following 3D subclassification prior to 3D-refinement, and all 3D classifications were carried out with a T-value of 4. This approach identified ~333,000 particles in classes with strong $CUL2^{LRR1}$ density. Following refinement of this subset of particles an improved mask was generated covering the entirety of the $CUL2^{LRR1}$ complex and the N-tier of MCM3. This mask was used to carry out an additional round of signal-subtraction and sub-classification resulting in ~310,000 replisome particles with stoichiometric $CUL2^{LRR1}$ association. These particles were then submitted for dataset-trained Bayesian polishing and re-extraction into a 430 Å box using an un-binned pixel size of 1.072 Å/pix, and CTF

parameters (beamtilt and trefoil correction, anisotropic magnification correction, and per-particle defocus and astigmatism CTF correction) were iteratively refined. A final round of 3D classification without alignment for the total complex was carried out and classes containing the full complement of replisome components were selected for, giving a total of ~232,000 particles. These particles were also refined in 3D using non-uniform refinement in cryoSPARC-2[17], yielding a reconstruction at 2.8 Å resolution (B-factor of -80 Å^2).

In order to improve the resolution of AND-1, an additional 3D classification step was carried out on the ~232,000 particles which yielded a reconstruction at 2.9 Å resolution (blue pathway Extended Data Fig. 8). This resulted in the selection of ~177,000 particles occupying classes containing strong secondary structure features present within AND-1. This subset was submitted for multibody refinement using a mask encompassing the AND-1 SepB domain trimer, CDC45 and GINS resulting in a reconstruction following postprocessing (B-factor of -40 Å^2) at 3.3 Å resolution. Refinement of 3D classes lacking AND-1 results in reconstructions lacking density attributed to the AND-1 HMG box in the region of the LRR1-CUL2-ELOB-ELOC interface.

Further focussed classification was carried out on the region encompassing the N-terminal half of CUL2, ELOB-ELOC and the LRR1 BC and CUL2 boxes (orange pathway Extended Data Fig. 8). Signal subtraction, followed by 3D classification without alignment, focussing upon this region resulted in the selection of ~88,000 particles in 3D classes displaying secondary structure features. Refinement of this subset of particles permitted the generation of an optimised mask covering the region in question. An additional round of signal subtraction and 3D classification without alignment resulted in a stack of ~54,000 particles. Multibody refinement of this subset yielded a reconstruction of the LRR1-ELOB-ELOC-CUL2 interface following postprocessing (B-factor of -80 Å^2) at 7.8 Å resolution.

In order to recover density comprising the entirety of CUL2/RBX1, the ~232,000 particle subset which yielded a reconstruction at 2.9 Å resolution, was re-extracted and downsampled to 4.288 Å/pixel; increasing the signal-noise for highly flexible regions (orange pathway Extended Data Fig. 8). Following refinement of the downsampled particles, a mask encompassing CUL2/RBX1 was generated in which the binary edge was extended by 40 Å with

a soft edge of 10 Å, ensuring all conformations of CUL2-RBX1 were included within the mask. Following signal subtraction and 3D classification without alignment, a variety of conformations were recovered. A single class representing ~39,000 particles was selected as representative of a single CUL2-RBX1 conformation based upon the presence of secondary structure features in the N-terminal half of CUL2. Following multibody refinement of this particle stack, a reconstruction for the CUL2-RBX1 body was obtained following postprocessing (B-factor of -100 $Å^2$) at 10.8 Å resolution.

During the initial stages of 3D classification, it was noted that ~625,000 particles did not contain $CUL2^{LRR1}$ stably bound (dark green pathway Extended Data Fig. 8). Following Bayesian polishing and CTF refinement, these particles were further classified in 3D resulting in the selection of ~415,000 particles classes lacking $CUL2^{LRR1}$. In order to be confident that this subset was homogenously lacking $CUL2^{LRR1}$, a previously used mask, covering the entirety of the $CUL2^{LRR1}$ complex and the N-tier of MCM3, was used to carry out signal subtraction and 3D classification without alignment. This enabled us to select ~388,000 particles in core-replisome classes lacking $CUL2^{LRR1}$. These particles were refined and postprocessed (B-factor of -30 $Å^2$) to a resolution of 2.8 Å. Interestingly a class containing ~ 87,000 particles, derived from the ~625,000 in which $CUL2^{LRR1}$ was not stably bound, was identified in which density for the LRR1 pleckstrin homology domain was present but lacking for the leucine-rich repeats. This class was refined and postprocessed (B-factor of -40 $Å^2$) to 3.8 Å resolution.

The sharpened maps described above, or the equivalent filtered maps generated during local resolution estimation by RELION, were used in subsequent model building. Maps were used either individually or after combination using Phenix combine_focused_maps.

Model building and refinement

S. cerevisiae replisome:SCF^{Dia2} complexes on dsDNA

Model building and refinement were performed iteratively using COOT[18], ISOLDE[19] and Phenix real-space refinement[20]. For Mcm2-7 and the C-tier-bound dsDNA, model building utilised maps derived separately for each of conformation I and II. For the remaining regions of the complex, no significant local differences were observed between conformations I and II enabling higher-resolution maps derived from combining more than one conformation to

be fitted and used to complete model building. A detailed description of map derivation is provided in the data processing section above.

For CMG, Ctf4, Tof1-Csm3 and the parental dsDNA, our previous model of CMG-CTM-Ctf4-DNA (PDB: 6SKL[7]) was used as a starting model and adjusted to density. For Tof1-Csm3 specifically, the region of the relevant map was isolated using the "zone" function in Chimera[21] to enable better fitting to each conformation prior to model building. Cα-backbone density was observed for Csm3 residues 42-45, allowing the model to be extended upstream of the DNA-binding motif to contact the concave LRR surface and C-terminal tail of Dia2 (see below). Furthermore, density was of sufficient resolution to build the Mcm5 winged helix (WH) domain using an I-TASSER[22-24] homology model as an initial starting point: the position of the Mcm5 WH was comparable to what has previously been reported[25], although our model reveals the details of its interaction with the non-catalytic module of Pol ε.

DNA was modelled as idealised B-form dsDNA and adjusted to density. The resolution of our cryo-EM reconstructions precluded our ability to determine the exact position of the replisome along the DNA substrate following translocation during sample preparation, compounded by the lack of a defined stalling position within the DNA. Therefore, the sequence was defined as poly(dG):poly(dC), with a short stretch modelled as poly(dT) where the density was sufficient to build only one strand of DNA, the rationale being to best fit the experimental cryo-EM density and reduce clashes with neighbouring regions of MCM.

The Pol2 and Dpb2 subunits of the non-catalytic module of Pol ε were modelled using the prior structure of CMG-Pol ε (PDB: 6HV9[25]) as a starting model. The improved resolution of our data enabled extension or rebuilding of several regions of Pol2 and Dpb2 (exemplified by regions of Pol2 in the vicinity of residues 1934-1975). For the Dpb2 N-terminal domain (residues 7-94), model building was aided by comparison to the equivalent region of the human complex[8].

With regard to SCF^{Dia2}, the resolution of our density enabled *de novo* model building of the Dia2 F-box (residues 211-247) and LRR (residues 248-716) domains, as well as the C-terminal

tail (residues 717-732). Model building of the F-box domain was aided by I-TASSER homology models[22-24] of the Dia2 F-box and comparison to other F-box proteins (e.g. PDB: 1LDK[26]). To model the Dia2 LRR domain and C-terminal tail, a combination of higher-resolution density for the C-terminal regions of Dia2, comparison to I-TASSER homology-based models[22-24] and Jpred secondary-structure[27] predictions, identification of density corresponding to specific bulky side-chains, continuity with the upstream F-box domain and identification of the characteristic LRR consensus motif[28] enabled model building of the complete LRR domain and C-terminal tail, with the exception of two disordered regions (residues 380-419 and 675-681).

The resolution of the density contributed by Skp1 was insufficient to enable *de novo* model building for the majority of the protein; however, a prior crystal structure of Skp1 from budding yeast (PDB: 1NEX[29]) could be unambiguously fitted to our density. Furthermore, the Skp1 C-terminus (absent from the crystal structure) could be modelled based on our density, aided by the prior crystal structure of the human Skp1:Skp2 complex (PDB: 1FQV[30]).

Finally, the resolution of our map was sufficient to resolve α-helices belonging to the cullin repeats of Cdc53. This allowed unambiguous rigid-body fitting of a crystal structure previously determined for the human CUL1-RBX1 subcomplex (homologous to Cdc53-Hrt1). However, as no structures exist for the yeast homologues, these subunits were omitted from our final models.

During model building, a final round of Phenix real-space-refinement using the input model as a reference to generate restraints with sigma=0.1 and global minimisation with nonbonded_weight=2000 and weight=0.5 was particularly useful for limiting the clash score. Both during and following completion of model building/refinement, model validation was performed using the MolProbity server[31], Phenix validation[20] and the wwPDB OneDep validation server[32]. Model-to-map FSCs were plotted using Xmipp[33] having generated a model-map using EMAN pdb2mrc[34] and removing solvent density from the relevant full- and half-maps using multiplication in RELION relion_image_handler[35].

***S. cerevisiae* replisome:SCF^{Dia2} complexes in the absence of DNA**

The model for the complex assembled on dsDNA (described above) was adjusted to cryo-EM density for the sample lacking DNA using ISOLDE[19], enabling comparison of the MCM:Dia2 interface (see Fig. 1g).

***H. sapiens* replisome:CUL2^{LRR1} complexes on 3'-flap DNA**

Model building and refinement were performed iteratively using COOT[18], ISOLDE[19] and Phenix real-space refinement[20].

For CMG, TIMELESS-TIPIN, AND-1, Pol ε^{nonCat} and DNA, our previous model of the core-human replisome[8] was used as a starting point for model building. The fit-to-density was optimised in ISOLDE and regions for which the model correlated poorly with the density were manually re-built in COOT. These included the N-terminus and residues 164-174 of MCM3 and the ZnF of MCM5.

The resolution of LRR1 enabled *de novo* model building for its pleckstrin homology domain (residues 1-116), leucine-rich repeats (LRRs) 3-8 (residues 203-305) and some of the flexible linker between them (residues 130-139). Due to the weaker density for LRRs 1-2 (residues 139-202) an I-TASSER[22-24] generated homology model for this region was rigid-body docked into the density and the fit optimised using ISOLDE and COOT. Analysis of the high-resolution density for LRR-9 enabled us to determine the sequence-from-density for this region. This permitted the building of both LRR-9 and the C-terminal capping beta sheet (residues 370-397) *de novo* with the assistance of bulky side-chains such as H377. At this stage the crystal structure of CUL2-RBX1-ELOB-ELOC (PDB:5N4W[36]) was docked into the remaining density of the multi-body map focussed on this region. The fit was unambiguous and was guided by secondary structure elements. Furthermore, in this conformation, the C-terminal half of CUL2 and RBX1 fit with high confidence into the multi-body map focussing on this region alone. Following this rigid body docking protocol, it was reasoned that the remaining unmodelled density in the vicinity of LRR1 must represent its CUL2- and BC-boxes. Into this density, unconnected poly-Ala α-helices were built, and their arrangement compared to the structure of the VHL-box (PDB:4JGH[37]), subsequently identifying the region of LRR1 comprising the BC-

box. These secondary structure elements were then connected manually in COOT and an additional C-terminal helix built into density following secondary structure predication. Additional density that could not easily be attributed to unmodelled regions of LRR1 or ELOB-ELOC still remained, into which the structure of the AND-1 HMG-box (PDB:2D7L) was docked with high confidence, guided by secondary structure elements.

Regions of the core replisome interacting with LRR1 were subsequently refined in COOT and ISOLDE. These regions include the MCM3 residues 152-158 which form a beta strand at the primary interface with the LRR1 LRRs, in addition to regions of the TIMELESS helical repeats 1-3 and the ZnFs of MCM6 and MCM2 that contact the LRR1 PH domain. A final round of real-space-refinement using the input model as a reference to generate restraints with sigma=0.1 and global minimisation with nonbonded_weight=2000 and weight=0.5 was carried out. Model validation was carried out using the MolProbity server[31], Phenix validation[20]and the wwPDB OneDep validation server[32]. Model-to-map FSCs were plotted using Xmipp[33] having generated a model-map using EMAN pdb2mrc[34] and removing solvent density from the relevant full- and half-maps using multiplication in RELION relion_image_handler[35].

***In vitro* replication-ubiquitylation assays**

Mcm2-7 loading and DDK phosphorylation was performed by incubating 6 nM 3.2 kb plasmid DNA template (pBS/ARS1WTA), 5-10 nM ORC, 20 nM Cdc6, 40 nM Cdt1·Mcm2-7 and 20 nM DDK in 25 mM Hepes-KOH (pH 7.6), 100 mM KOAc, 0.02% NP-40-S, 0.1 mg/mL BSA, 1 mM DTT, 10 mM $Mg(OAc)_2$ and 5 mM ATP at 30°C for 10 min.

Separate buffer and replication protein mixtures were next added sequentially to the Mcm2-7 loading mixture. 10 µL of the Mcm2-7 loading mixture was generally used per sample and this was typically diluted 2-fold in the final reaction. The final replication reaction contained 25 mM Hepes-KOH (pH 7.6), 100 mM KOAc, 0.02% NP-40-S, 0.1 mg/ml BSA, 1 mM DTT, 10 mM $Mg(OAc)_2$, 3.75 mM ATP, 30 µM dATP-dCTP-dGTP-dTTP, 33 nM γ-[^{32}P]-dCTP, 400 µM CTP-GTP-UTP, 20 µM creatine phosphate, 50 µg/mL creatine phospho-kinase, 6 µM ubiquitin, 20 nM S-CDK, 30 nM Dpb11, 8 nM GINS, 40 nM Cdc45, 30 nM Pol ε, 5 nM Mcm10, 5 nM RFC, 20 nM PCNA, 20 nM Top1, 20 nM Pol α-primase, 6.25 nM Sld3-7, 40 nM Ctf4, 50 nM RPA, 10 nM

Tof1-Csm3, 40 nM Mrc1, 50 nM Sld2 and 5 nM Pif1 (unless otherwise indicated). Pol δ (2.5 nM), Fen1 (10 nM) and Cdc9 ligase (20 nM) were included as indicated. The extra contribution from protein storage buffers to the final reaction was approximately 22 mM chloride and 50-60 mM acetate, and the corresponding potassium counter-ions.

The replication step was routinely conducted at 30°C for 20 min. 30 nM Uba1, 15 nM Cdc34 and 2 nM SCF^{Dia2} were added after the replication step, and the incubation continued at 30°C for a further 20 min. Ubiquitylation reactions were stopped by the addition of KOAc to 700 mM. Next, plasmid DNA was digested by addition of 125 U Pierce Universal Nuclease (ThermoFisher Scientific, 88702) and incubation on ice for 30 min. Each sample was then incubated for 30 min at 4°C with 10 µL magnetic Dynabeads M-270 Epoxy (Life Technologies) that had been coupled to antibodies raised against Sld5. After the incubation, protein complexes bound on antibody-coupled magnetic beads were washed twice with 190 µL of buffer containing 25 mM Hepes-KOH (pH 7.6), 700 mM KOAc, 0.02 % NP-40-S, 0.1 mg/mL BSA, 1 mM DTT, 10 mM $Mg(OAc)_2$ (Wash buffer / 700 mM KOAc). The bound proteins were then eluted by the addition of SDS-PAGE sample loading buffer and boiling for 5 min at 95°C.

Immunoprecipitation of TAP-Sld5 following G1-arrest

Yeast cells were grown at 30°C in YP medium supplemented with 2% glucose (YPD). To synchronize cells in G1-phase, α-factor mating pheromone (Pepceuticals Limited) was added to log-phase cell cultures (cell density: 0.7×10^7 cells/ml) to a final concentration of 7.5 µg/mL. After 1 h, additional aliquots of 2.5 µg/mL (final concentration) α-factor were added every 15 min until ~90% cells were unbudded and schmooing. To release from G1-phase arrest, cells were washed twice with fresh medium lacking α-factor, and then resuspended in medium lacking α-factor to the original cell density.

To prepare yeast whole cell extracts from frozen yeast cells, 250 ml of a cell culture was first pelleted at 200*g* for 3 min. Cells were washed once in 50 mL of lysis buffer (100 mM Hepes-KOH (pH 7.9), 50 mM potassium acetate, 10 mM $Mg(OAc)_2$, 2 mM EDTA) and then resuspended in three pellet volumes of lysis buffer supplemented with 2 mM sodium fluoride, 2 mM sodium β-glycerophosphate pentahydrate, 1 mM DTT, 1% Protease Inhibitor Cocktail

(P8215, Sigma-Aldrich), and 1X Complete Protease Inhibitor Cocktail (Roche; a 25X stock solution was made by dissolving 1 tablet in 1 ml water) before freezing dropwise in liquid nitrogen.

~2-2.5 g of frozen yeast cells were ground in a SPEX SamplePrep 6780 Freezer/Mill (2 x 2 min cycles on setting 14). After thawing, the thawed extract was supplemented with 0.25 volumes of glycerol mix buffer containing 100 mM Hepes-KOH (pH 7.9), 50 % glycerol, 300 mM potassium acetate, 10 mM $Mg(OAc)_2$, 2 mM EDTA, 0.5% NP-40, 1 mM DTT and the protease and phosphatase inhibitors at the concentrations mentioned above. Chromosomal DNA was then digested by addition of 400 U/mL Pierce Universal Nuclease (123991963, Fisher) and incubation for 30 min at 4°C. Insoluble cell debris was pelleted in two high-speed centrifugation steps (at 25000 × g for 30 min and then at 100000 × g for 1 h).

Samples of the recovered cell extracts (typically 50 µL) were removed the remaining fraction of each cell extract (~2 ml) was then split into two aliquots, each of which was incubated with 1.7×10^9 magnetic beads (Dynabeads M-270 Epoxy; 14302D, Life Technologies) that had been coupled to rabbit immunoglobulin G (IgG) (S1265, Sigma-Aldrich) for 2 h at 4°C. After the incubation, protein complexes bound on antibody-coupled magnetic beads were washed four times with 1 ml of wash buffer (100 mM Hepes-KOH (pH 7.9), 100 mM KOAc, 10 mM $Mg(OAc)_2$, 2 mM EDTA, 0.1% IGEPAL CA-630, 2 mM sodium fluoride, 2 mM sodium β-glycerophosphate pentahydrate, 1% Protease Inhibitor Cocktail (Sigma-Aldrich), and 1X Complete Protease Inhibitor Cocktail (Roche)). The bound proteins were then eluted by heating at 95°C for 5 min in 50 µL SDS-PAGE sample loading buffer.

Flow Cytometry

For each sample, 10^7 yeast cells were harvested and fixed by resuspension in 1 ml of 70% ethanol. Subsequently, 3 ml of 50 mM sodium acetate and 50mg of RNase A was added to 150 µL of fixed cells, followed by incubation at 37°C for 2 h. The cells were then pelleted and proteins degraded by incubation at 37°C for 30 min in 500 µL of 50 mM HCl containing 2.5 mg of Pepsin. Finally, cells were pelleted and then re-suspended in 1 ml of 50 mM sodium citrate containing 2 mg of propidium iodide. Samples were sonicated and then analysed in a FACSCanto II flow cytometer (Becton Dickinson). The data were analysed with FlowJo™ v10.8

Software (BD Life Sciences). For details of gating strategy and assignment of the G1 peak see Supplementary Fig. 2.

Dia2 mutant growth assays

Yeast strains were constructed and manipulated by standard genetic techniques. Mutagenesis of *DIA2* at the endogenous locus was done by first cloning full-length *DIA2* harbouring the 13A mutations upstream of *URA3* in plasmid pKL506 (Labib laboratory). The resultant *DIA2-URA3* cassette was amplified from plasmid pTDK48 (see supplementary table 2) by PCR and then used to replace an endogenous copy of *DIA2* by transformation into a yeast diploid. Mutant haploid yeast strains were then isolated by tetrad dissection of the resultant heterozygous diploid strain.

For spot-dilution assays, 10-fold dilutions of each yeast strain were spotted onto YPD-agar plates and incubated at the indicated temperature for the indicated time.

***In vitro* CMG ubiquitylation assays (off DNA)**

Reactions (8-10 μL volume) containing 15 nM CMG, 30 nM Uba1, 15 nM Cdc34, 1 nM SCF^{Dia2}, 30 nM Ctf4, 30 nM Pol ε and 45 nM Mrc1 were assembled on ice in 25 mM Hepes-KOH (pH 7.6), 75 mM KOAc, 0.02% NP-40-S, 0.1 mg/mL BSA, 1 mM DTT, 10 mM $Mg(OAc)_2$, 6 μM ubiquitin and 5 mM ATP. Protein storage buffers typically contributed approximately 50 mM acetate, and the corresponding potassium counter-ions, to the final reaction. Ubiquitylation reactions were incubated at 30°C for 20 min and stopped by the addition of SDS-PAGE sample loading buffer.

Interaction of SCF^{Dia2} with CMG with purifed proteins

20 μL reactions containing 15 nM CMG, 30 nM Uba1, 15 nM Cdc34, 10 nM SCF^{Dia2} and 30 nM Pol ε were assembled in 25 mM Hepes-KOH (pH 7.6), 75 mM potassium acetate, 0.02% NP-40-S, 0.1 mg / ml BSA, 1 mM DTT, 10 mM $Mg(OAc)_2$, 6 μM ubiquitin and 5 mM ATP. Mrc1 (45 nM) and Ctf4 (30 nM) were added as indicated and the reactions incubated on ice for 10 min. Input samples (typically 5 μL in volume) were removed and the remainder of each sample was then incubated for 30 min at 4°C with 3.75 μL magnetic beads (Dynabeads M-270 Epoxy; 14302D, Life Technologies) that had been coupled to anti-Sld5 antibody. After the incubation,

protein complexes bound on beads were washed twice with 190 µL of Wash buffer / 150 mM KOAc (25 mM Hepes-KOH (pH 7.6), 150 mM potassium acetate, 0.02 % NP-40-S, 0.1 mg / ml BSA, 1 mM DTT, 10 mM $Mg(OAc)_2$). The bound proteins were then eluted by the addition of SDS-PAGE sample loading buffer and boiling for 5 min at 95°C.

In vitro Ctf4 ubiquitylation assays

Reactions containing 30 nM Uba1, 15 nM Cdc34, 5 nM SCF^{Dia2} and 30 nM Ctf4 were assembled on ice in 25 mM Hepes-KOH (pH 7.6), 75 mM KOAc, 0.02% NP-40-S, 0.1 mg/mL BSA, 1 mM DTT, 10 mM $Mg(OAc)_2$, 6 µM ubiquitin and 5 mM ATP. Ubiquitylation reactions were incubated at 30°C for 20 min and stopped by the addition of SDS-PAGE sample loading buffer.

DNA replication assay on a forked DNA template

To prepare the 9.7 kbp primed forked DNA template for replication assays, oligonucleotides MT096 - (5'Phos)GCTATGTGGTAGGAAGTGAGAATTGGAGAGTGTGTTTTTTTTTTTTTTTTTTTTTTTTTTTTTTTTTTTTTTTTGAGGAAAGAATGTTGGTGAGGGTTGGGAAGTGGAAGGATGGGCTCGAGAGGTTTTTTTTTTTTTTTTTTTTTTTTTTTTTTTTTTTTTT, JY195 – CCTCTCGAGCCCATCCTTCCACTTCCCAACCCTCACC and JY197 -TTTTTTTTTTTTTTTTTTTTCACACTCTCCAATTCTCACTTCCTACCACAT (Integrated DNA Technologies) were annealed and ligated to SapI-linearised ZN3[3]. The ligation was performed for 16 h at 16°C in a 300 µL reaction containing 1X T4 ligase buffer (New England Biolabs), 5 mM $MgCl_2$, 15 nM SapI-linearised ZN3, 375 nM annealed replication fork and 7,000 units T4 DNA ligase. The ligation was stopped by addition of EDTA to 30 mM and deproteinised by addition of SDS and proteinase K (New England Biolabs) to 0.25% and 0.5 mg/ml respectively followed by incubation at 37°C for 20 min. Excess fork oligonucleotides were removed by gel filtration through a 0.7 x 50 cm Sepharose-4B (Sigma) column developed in 5 mM Tris-Cl (pH 7.2), 0.1 mM EDTA. Peak fractions were pooled, ethanol precipitated and stored in 10 mM Tris-Cl (pH 7.2), 1 mM EDTA.

Replication assays were performed as follows. Yeast CMG was prebound to the forked DNA template in a buffer (CMG bind) containing 25 mM Hepes-KOH (pH 7.6), 100 mM potassium glutamate, 10 mM $Mg(OAc)_2$, 0.01% NP40, 1 mM DTT, 0.1 mg/ml BSA, 0.05 mM AMP-PNP, 2 nM forked-ZN3 template and 50 nM CMG. After a 5 min incubation at 30°C, CMG bind was mixed ~1:1 with reaction mix to give a reaction buffer containing 25 mM Hepes-KOH (pH 7.6),

100 mM potassium glutamate, 10 mM $Mg(OAc)_2$, 0.01% NP40, 1 mM DTT, 0.1 mg/ml BSA, 0.025 mM AMP-PNP, 1 nM forked-ZN3 template and 25 nM CMG, 20 nM Pol ε, 20 nM RFC, 20 nM PCNA, 20 nM Pol α, 10 nM Mrc1, 20 nM Tof1-Csm3, 20 nM Ctf4 and 30 μM dA/dCTP. After 5 min incubation at 30°C to enable replisome assembly, reactions were initiated with 10X Start buffer containing (final concentrations) 3 mM ATP, 200 μM C/U/GTP, 30 μM dG/dTTP, 100 nM RPA and 33 nM α-[^{32}P]-dCTP. Reactions were quenched by addition of EDTA to 25 mM and processed as described previously[2].

Immunoblotting

For detection of polyubiquitylated Mcm7 and Ctf4, proteins were resolved by SDS-PAGE using NuPAGE Novex 3 - 8% Tris-Acetate gels with NuPAGE Tris-Acetate SDS buffer at 200V for 70 min. For all other samples, NuPAGE Novex 4 - 12% Bis-Tris gels with NuPAGE MOPS SDS buffer were used at 200V for 50 min. Resolved proteins were transferred onto a nitrocellulose iBlot membrane (Invitrogen) with the iBlot Dry Transfer System (Invitrogen). All polyclonal primary antibodies used in this study were raised in sheep against the indicated yeast proteins and used at the following dilutions: α-Cdc45 (1:2000), α-Cdc53 (1:500; Santa Cruz Biotechnology, sc-50444), α-Ctf4 (1:10,000), α-Hrt1 (1:2000), α-Mcm5 (1:2000), α-Mcm6 (1:2000), α-Mcm7 (1:2000), α-Psf1 (1:1000), α-Skp1 (1:500; Santa Cruz Biotechnology, sc-5328), α-Sld5 (1:1000). Peroxidase anti-peorxidase (Sigma, P1291) was used to detect TAP-Sld5 (1:5000 dilution). A conjugate to horseradish peroxidase of anti-sheep IgG from donkey (Sigma, A3415) was used as a secondary antibody (1:10,000 dilution) before the detection of chemiluminescent signals on Hyperfilm ECL (Amersham, GE Healthcare) using ECL Western Blotting Detection Reagent (GE Healthcare).

Structural analysis and visualisation

All figures of cryo-EM density maps and models were produced using ChimeraX[38] with the exception of cryo-EM density coloured by local resolution, produced using Chimera[21]. Calculations of buried surface area were performed using PDBePISA[39]. Fourier shell correlation curves generated using RELION[9,35] were plotted using Prism 8.0.0 for OS X, GraphPad Software, San Diego, California USA, www.graphpad.com. Root-mean-square deviation (RMSD) values were calculated using the PyMOL Molecular Graphics System

(Version 2.4.1 Schrödinger, LLC) for the regions of MCM at the interface with SCF^{Dia2} (Mcm3 α1, α5 and the ZnF, the Mcm5 ZnF and the Mcm7 N-terminus).

Strain	Genotype
yTDK29	*MATa ade2-1 ura3-1 his3-11,15 trp1-1 leu2-3,112 can1-100*<br>*bar1Δ::hphNT*<br>*pep4Δ::kanMX*<br>*leu2::pRS305-Hrt1/Cdc53 codon optimised*<br>*ura3::pRS306-Skp1/PrA-3Tev-Dia2 3A codon optimised*
yTDK30	*MATa ade2-1 ura3-1 his3-11,15 trp1-1 leu2-3,112 can1-100*<br>*bar1Δ::hphNT*<br>*pep4Δ::kanMX*<br>*leu2::pRS305-Hrt1/Cdc53 codon optimised*<br>*ura3::pRS306-Skp1/PrA-3Tev-Dia2 4A codon optimised*
yTDK31	*MATa ade2-1 ura3-1 his3-11,15 trp1-1 leu2-3,112 can1-100*<br>*bar1Δ::hphNT*<br>*pep4Δ::kanMX*<br>*leu2::pRS305-Hrt1/Cdc53 codon optimised*<br>*ura3::pRS306-Skp1/PrA-3Tev-Dia2 6A codon optimised*
yTDK32	*MATa ade2-1 ura3-1 his3-11,15 trp1-1 leu2-3,112 can1-100*<br>*bar1Δ::hphNT*<br>*pep4Δ::kanMX*<br>*leu2::pRS305-Hrt1/Cdc53 codon optimised*<br>*ura3::pRS306-Skp1/PrA-3Tev-Dia2 8A codon optimised*
yTDK33	*MATa ade2-1 ura3-1 his3-11,15 trp1-1 leu2-3,112 can1-100*<br>*bar1Δ::hphNT*<br>*pep4Δ::kanMX*<br>*leu2::pRS305-Hrt1/Cdc53 codon optimised*<br>*ura3::pRS306-Skp1/PrA-3Tev-Dia2 13A codon optimised*
yCPR126	*MATa ade2-1 ura3-1 his3-11,15 trp1-1 leu2-3,112 can1-100*<br>*dia2-13A (ura3)*
yTDK38	*MATa ade2-1 ura3-1 his3-11,15 trp1-1 leu2-3,112 can1-100*<br>*dia2-13A (ura3)*<br>*NTAP2-SLD5 (kanMX)*<br>*pep4Δ::ADE2*
yJY97	*MATa ade2-1 ura3-1 his3-11,15 trp1-1 leu2-3,112 can1-100*<br>*bar1Δ::Hyg*<br>*pep4Δ::KanMX*<br>*trp1::TRP1pRS304Mcm4, Mcm5*<br>*ura3::URA3pRS306/Mcm2, CBP-TEV Mcm3*<br>*leu2::LEU2pRS305/Mcm6, Mcm7*<br>*his::HIS3pRS303Cdc45iFlag2*<br>Ade2::PatMX3-vJY110 Psf2, Psf3<br>lys2::NAT-vJY109 Psf1, Sld5
yJY197	*MATa ade2-1 ura3-1 his3-11,15 trp1-1 leu2-3,112 can1-100*<br>*bar1Δ::Hyg*<br>*pep4Δ::KanMX*<br>*trp1::TRP1pRS304Mcm4, Mcm5*<br>*ura3::URA3pRS306/Mcm2, CBP-TEV Mcm3*<br>*leu2::LEU2pRS305/Mcm6, Mcm7*

	his3::HIS3pRS303Cdc45iFlag2<br>Ade2::PatMX3-vJY160 Psf2, Psf3, Psf1, Sld5
yJY231	*MAT*a *ade2-1 ura3-1 his3-11,15 trp1-1 leu2-3,112 can1-100*<br>*bar1Δ::Hyg*<br>*pep4Δ::KanMX*<br>*his3::HIS3pRS303-Cdc45iFlag2*<br>*Ade2::GINS-Pat*<br>*Ura3::URA3Mcm2/ CBP-TEV Mcm3-8A*<br>*trp1::TRP1pRS304Mcm4, Mcm5-5A*<br>*Leu2::LEUpRS305Mcm6, Mcm7*<br>*Ctf4-twin strep (Nat)*

Supplementary Table 1. *S. cerevisiae* strains constructed for this study.

Name	Protein(s) expressed	Vector details
DU70832	PrA-3TEV-Dia2 3A, Skp1	pRS based vector. PrA-3TEV-Dia2 3A and Skp1 under control of Gal1-10 promoter. Synthetic construct* cloned by Gibson assembly into pTDK3[5].
DU70833	PrA-3TEV-Dia2 4A, Skp1	pRS based vector. PrA-3TEV-Dia2 4A and Skp1 under control of Gal1-10 promoter. Synthetic construct* cloned by Gibson assembly into pTDK3[5].
DU70834	PrA-3TEV-Dia2 6A, Skp1	pRS based vector. PrA-3TEV-Dia2 6A and Skp1 under control of Gal1-10 promoter. Synthetic construct* cloned by Gibson assembly into pTDK3[5].
DU70835	PrA-3TEV-Dia2 8A, Skp1	pRS based vector. PrA-3TEV-Dia2 8A and Skp1 under control of Gal1-10 promoter. Synthetic construct* cloned by Gibson assembly into pTDK3[5].
DU70836	PrA-3TEV-Dia2 13A, Skp1	pRS based vector. PrA-3TEV-Dia2 13A and Skp1 under control of Gal1-10 promoter. Synthetic construct* cloned by Gibson assembly into pTDK3[5].
DU70831	Cdc34 C95K	pET28c based vector. Made by site-directed mutagenesis of pTDK7[5].
DU70837	Dia2 13A-URA3	Dia2 13A cloned XmaI / AscI into pKL506 (Labib laboratory).
vJY109	Psf1, Sld5	pRS based vector. Psf1 and Sld5 under control of Gal1-10 promoter. Nat-NT2 resistance marker and Lys2 nucleotides 3321-3799 for integration at Lys2 and selection with nourseothricin.

vJY110	Psf3, Psf2	pRS based vector. Psf2 and Psf3 under control of Gal1-10 promoter. Pat-MX4 resistance marker and Ade2 nucleotides 1040-1465 for integration at Ade2 and selection with glufosinate.
vJY154	LRR1	Synthetic construct** cloned BamHI / XbaI into pACEBac1
vJY155	2xFLAG-CUL2	Synthetic construct** cloned BamHI / XbaI into pACEBac1. MDYKDDDGDYKDDDG tag sequence at N-terminus.
vJY156	RBX1	Synthetic construct** cloned BamHI / XbaI into pACEBac1
vJY157	ELOB	Synthetic construct** cloned BamHI / XbaI into pACEBac1
vJY158	ELOC	Synthetic construct** cloned BamHI / XbaI into pACEBac1
vJY159	LRR1, 2xFLAG-CUL2, RBX1, ELOB, ELOC	pBig1c based vector for expression of $CUL2^{LRR1}$
vJY160	Psf1, Sld5, Psf3, Psf2	pRS based vector. Psf1, Sld5 and Psf2 and Psf3 under control of Gal1-10 promoters. Pat-MX4 resistance marker and Ade2 nucleotides 1040-1465 for integration at Ade2 and selection with glufosinate.
pMJB11	Mcm4 / Mcm5-5A (M182A,N185A,R187A,T189A, I244A).	pRS304-Mcm4-Gal1-10-$Mcm5^{M182A,N185A,R187A,T189A,I244A}$. Subclone $Mcm5^{M182A,N185A,R187A,T189A,I244A}$ (synthetic construct) using restriction sites BstBI/NsiI
pMJB13	Mcm2 / Mcm3-8A (P17A,D18A, F21A,K127A,R208A,H210A, Y211A,R224A)	pRS306-CBP-TEV-SgrAI-$Mcm3^{P17A,D18A,F21A,K127A,R208A,H210A,Y211A,R224A}$-

		Gal1-10-Mcm2. Subclone $Mcm3^{P17A,D18A,F21A,K127A,R208A,H210A,Y211A,R224A}$ (synthetic construct) using restriction sites AscI/AvrII

*codon optimized for expression in *S. cerevisiae*[2]
**codon optimized for expression in *E. coli* (Life Technologies)

Supplementary Table 2. Plasmids constructed for this study.

Supplementary Figure 1

Figure 2b

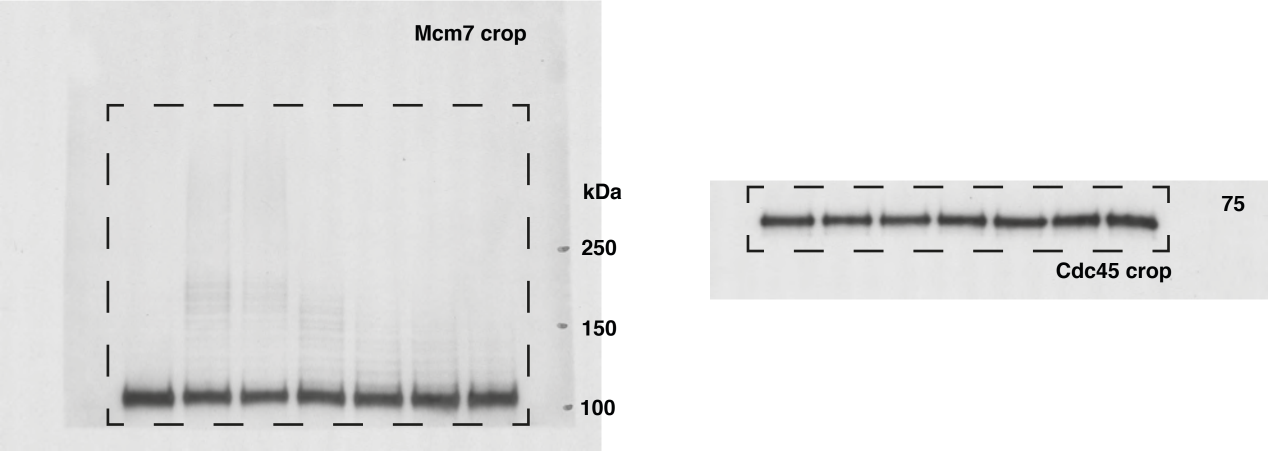

Figure 2c

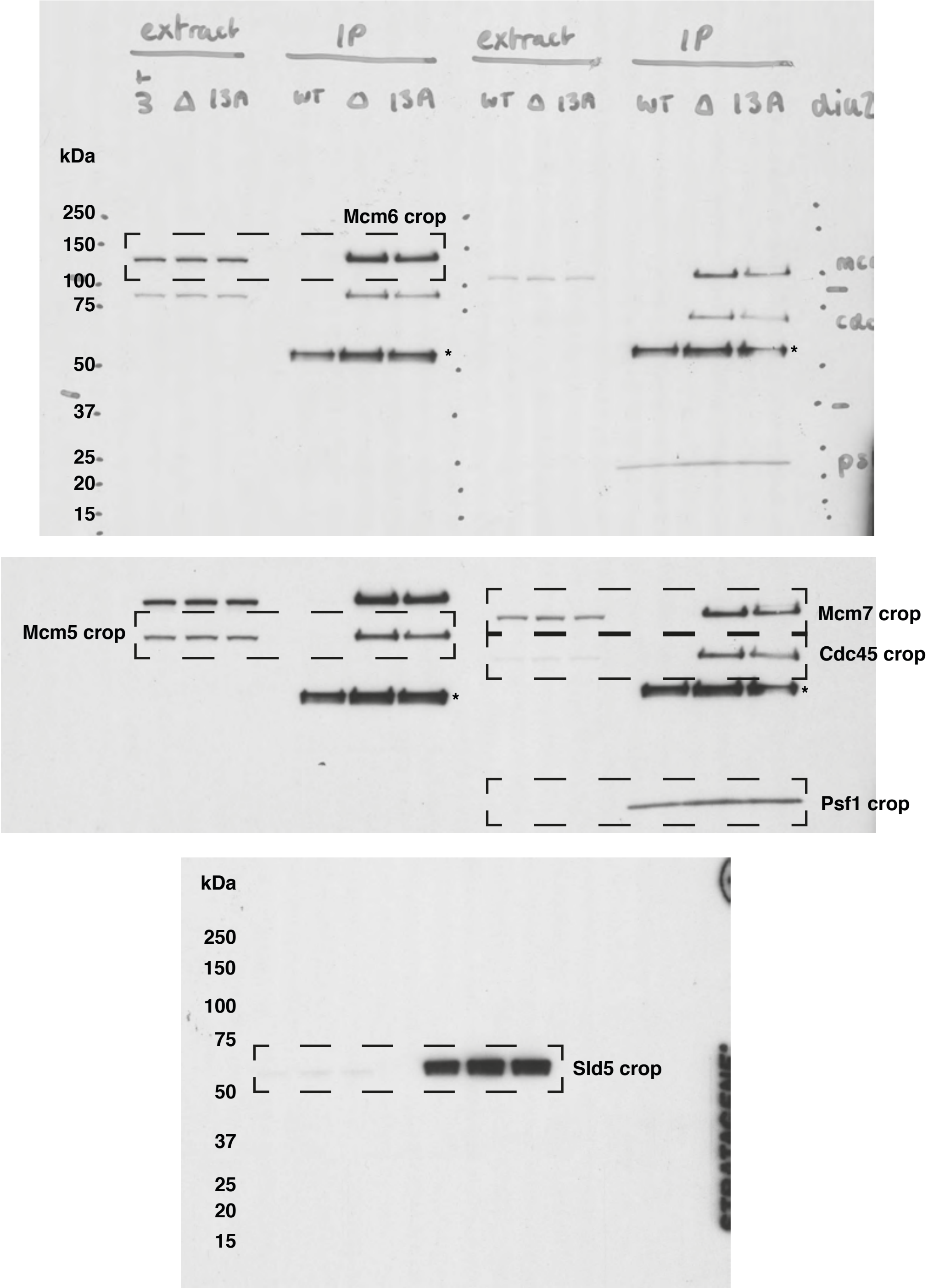

Extended Data Fig. 1b

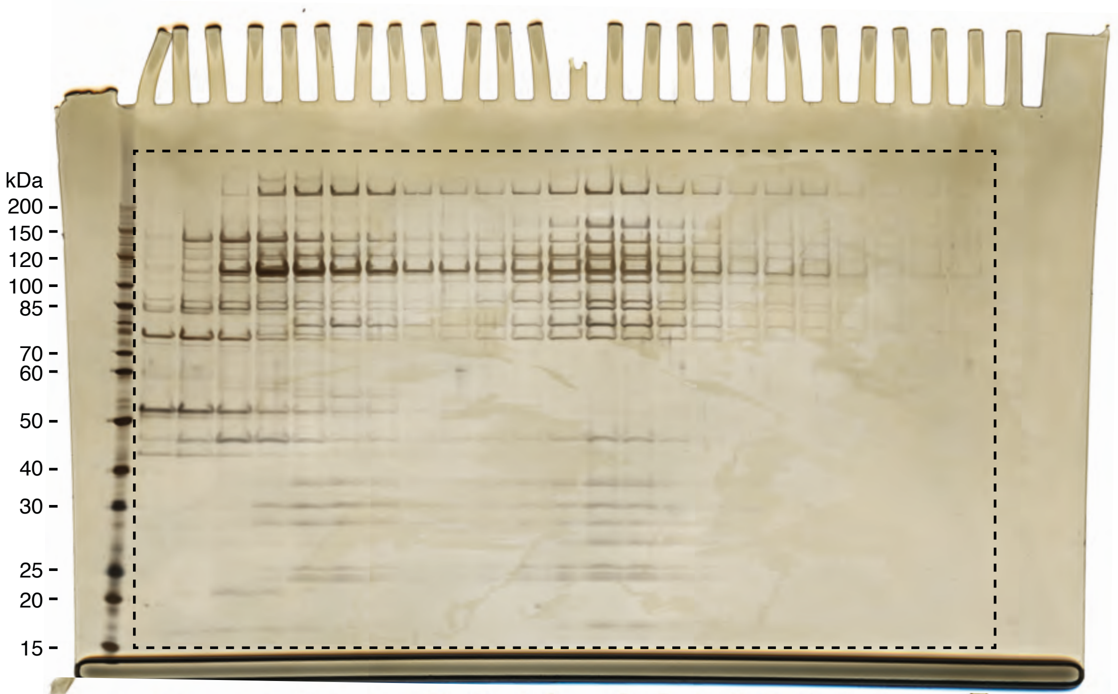

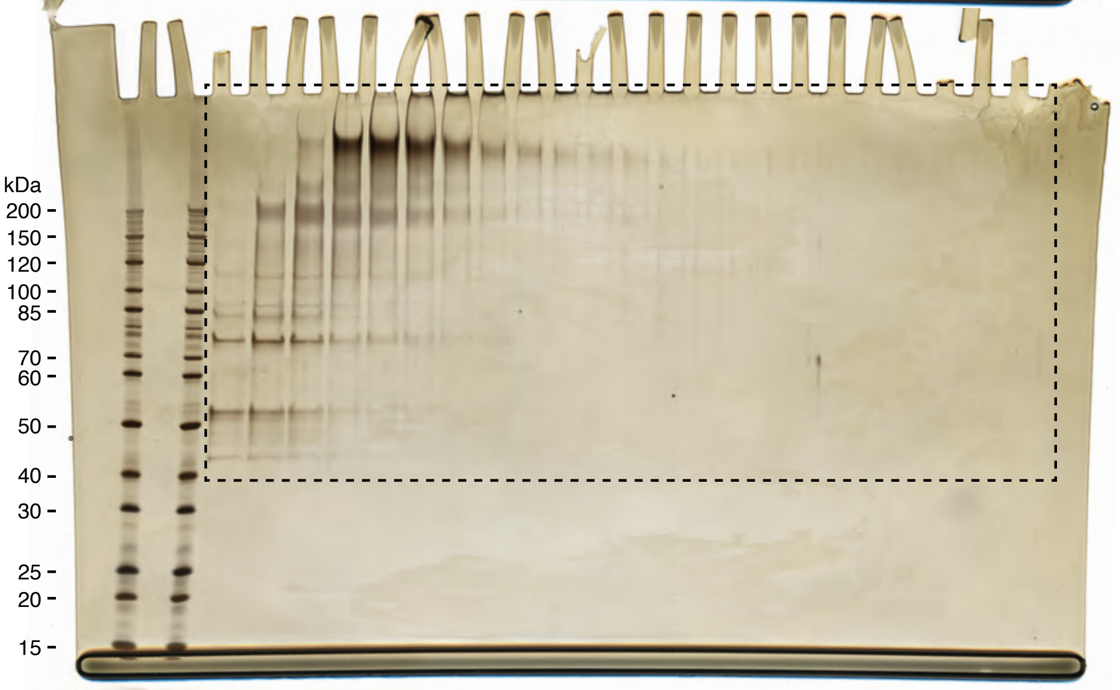

Extended Data Fig. 6a

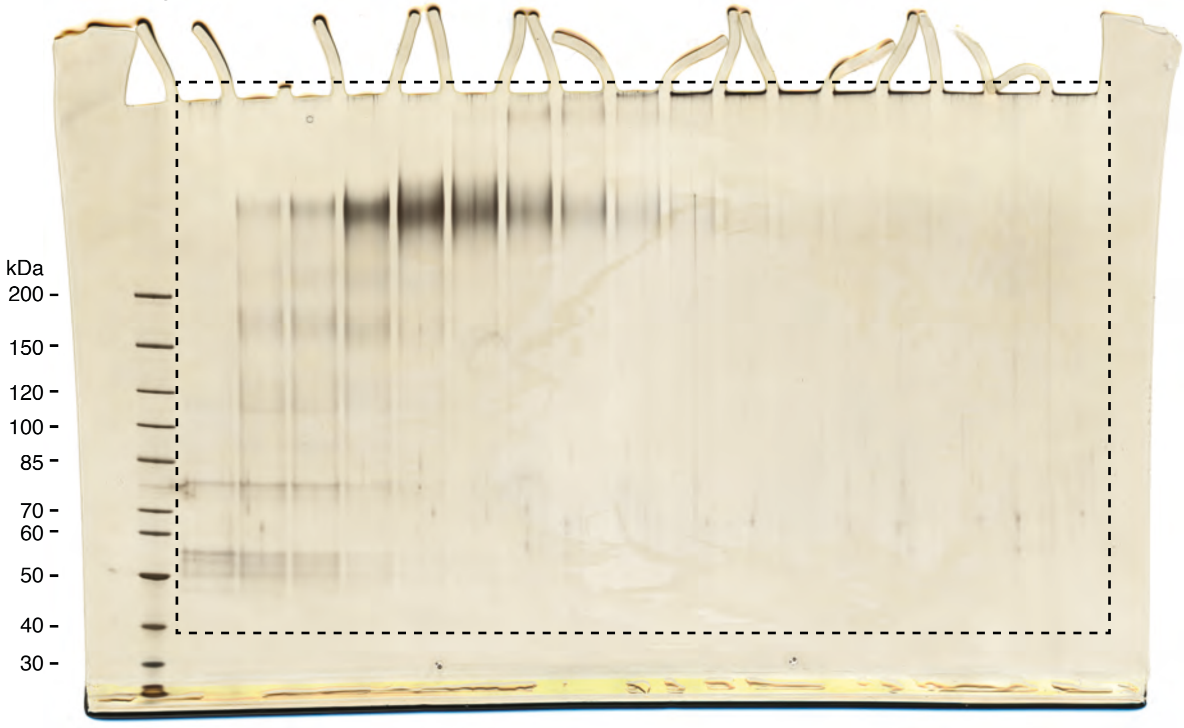

Extended Data Fig. 7a

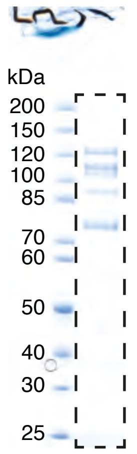

Extended Data Figure 7d

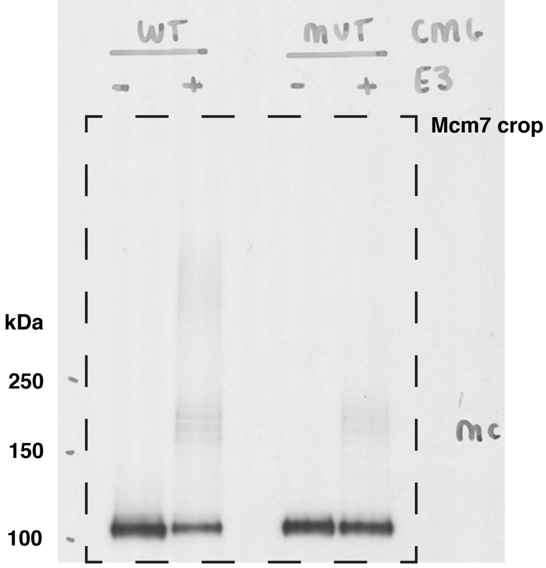

Extended Data Figure 7f

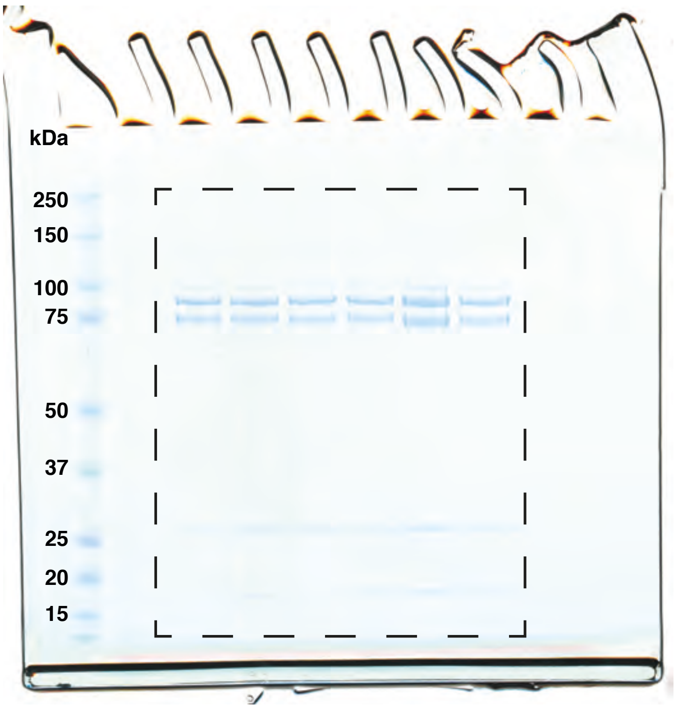

Extended Data Fig. 7c

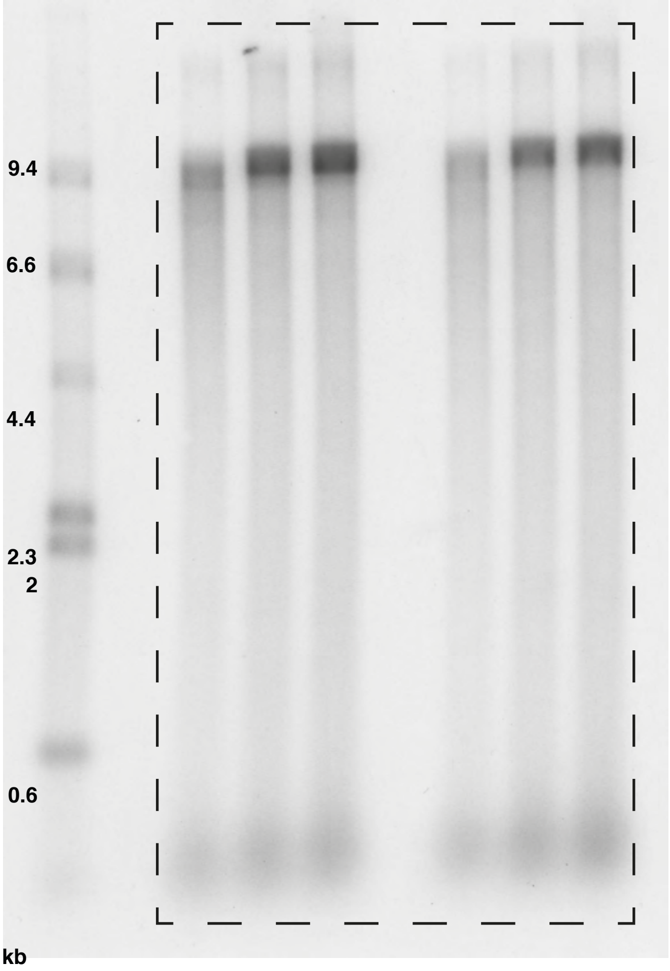

Extended Data Figure 7g

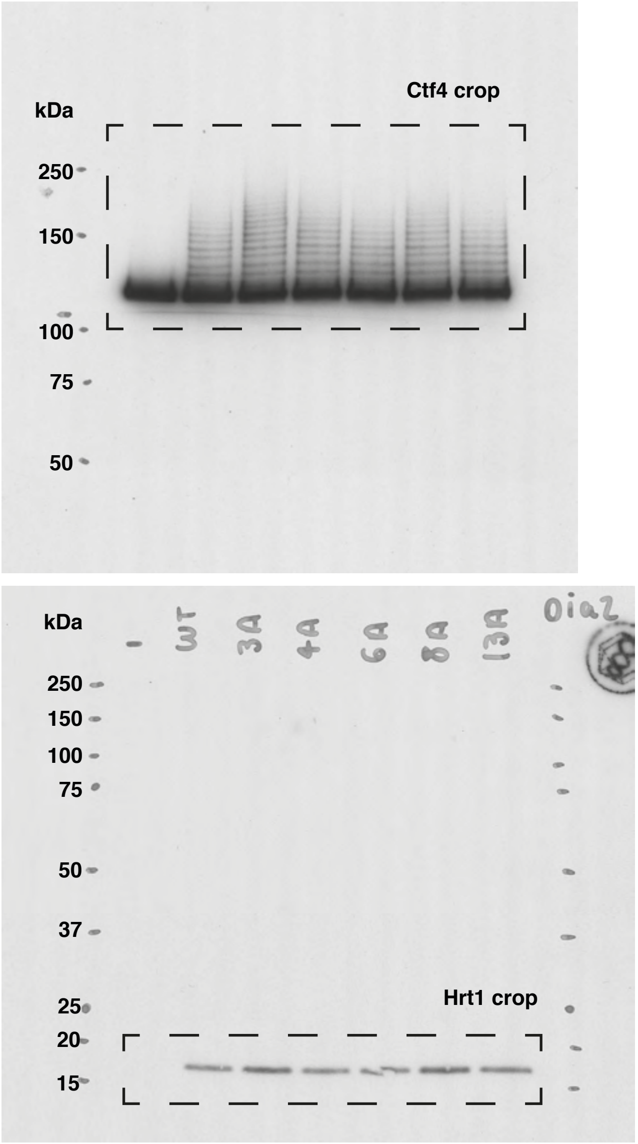

Extended Data Figure 7h.

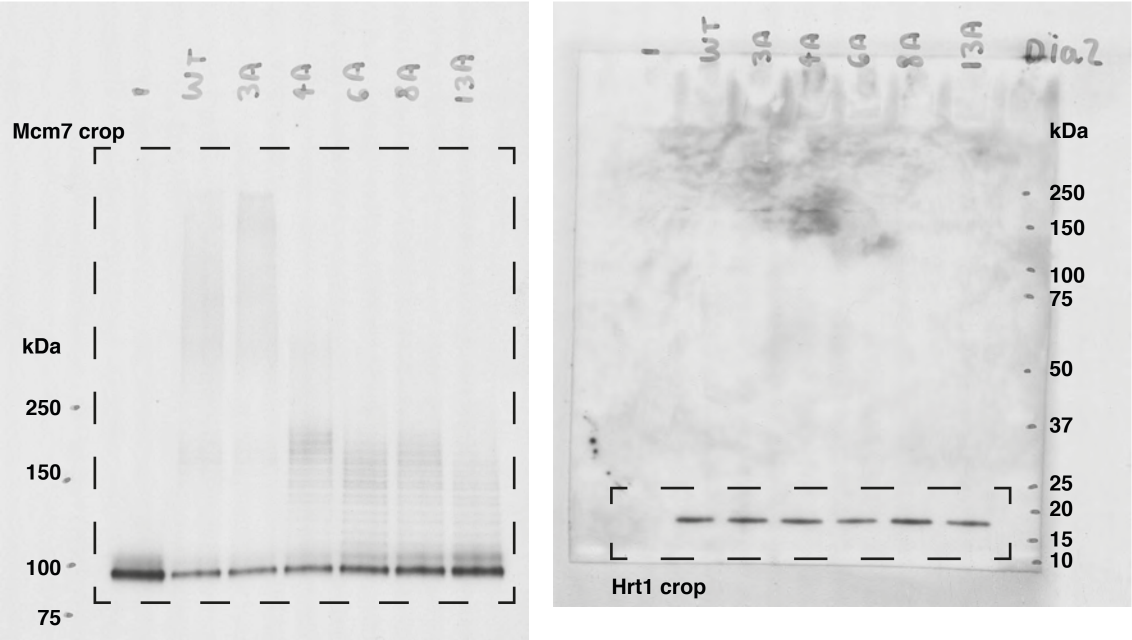

Extended Data Figure 9b.

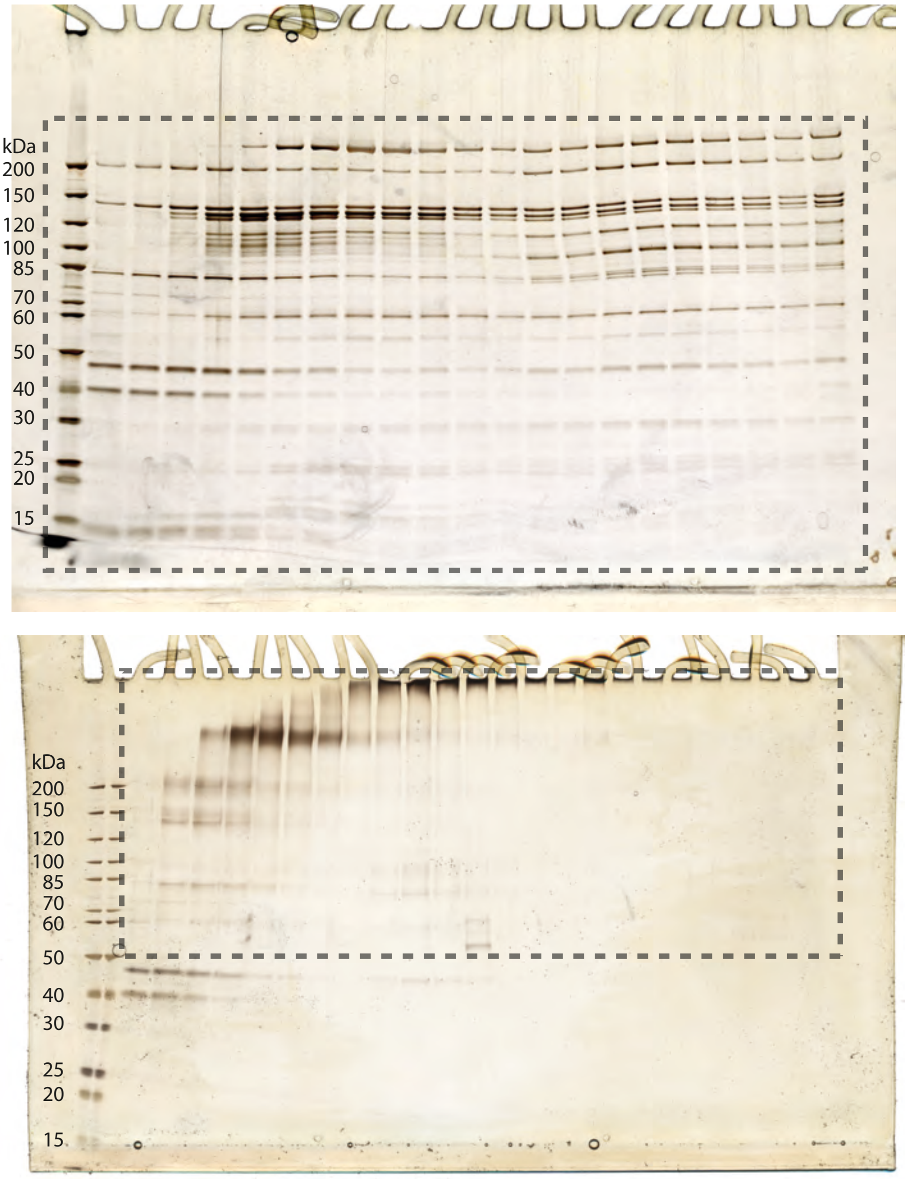

Extended Data Figure 11c.

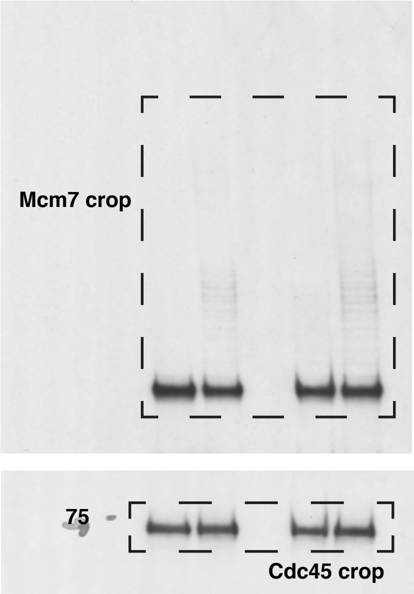

Extended Data Figure 11e.

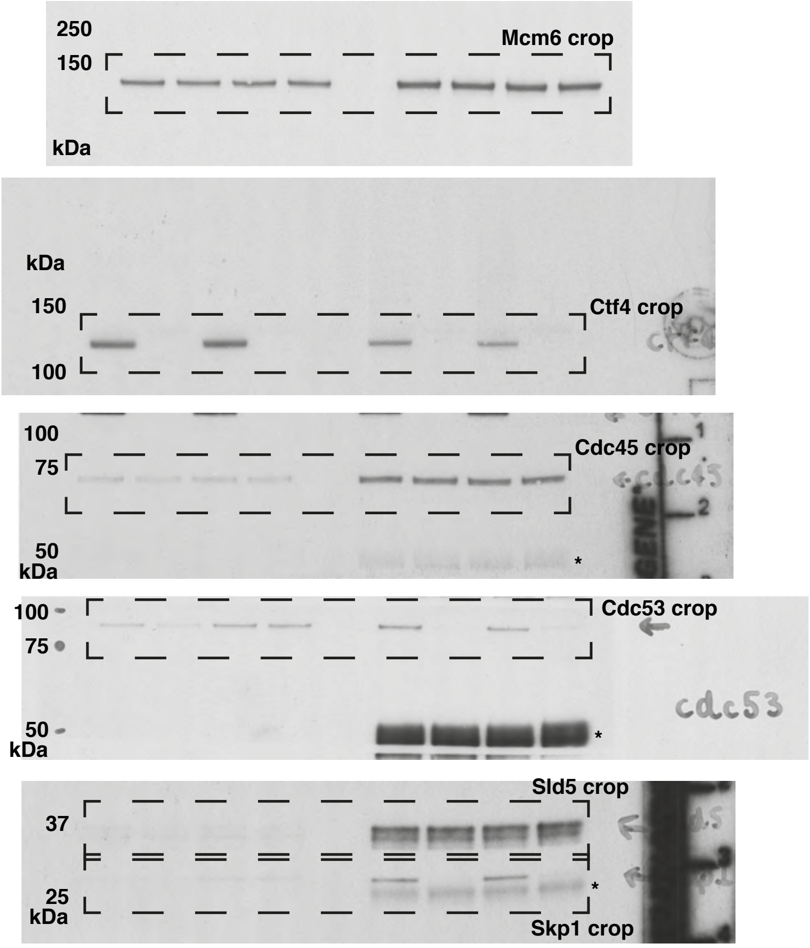

Supplementary Figure 2

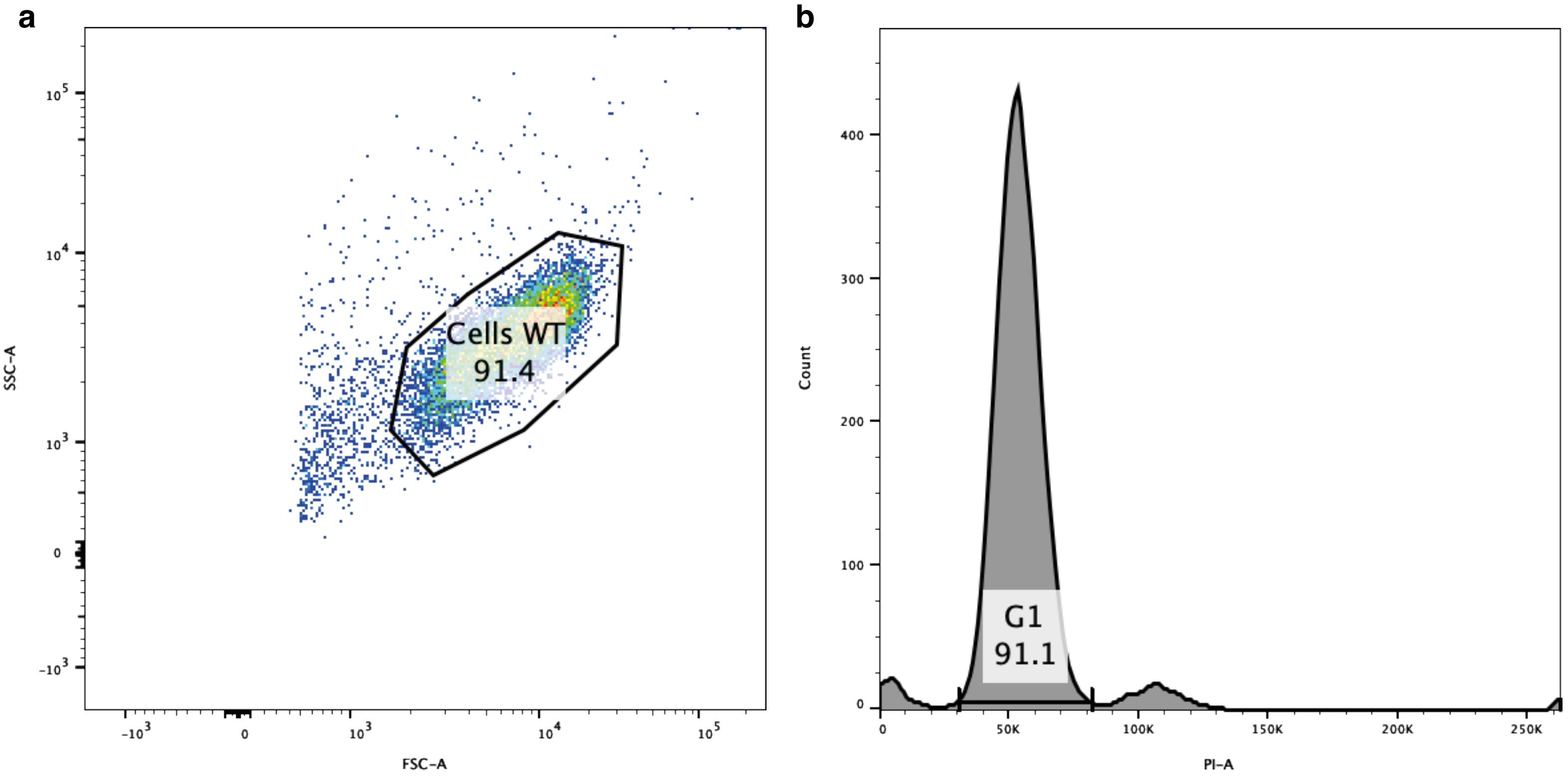

Supplementary Figure 1. Uncropped gel images. Figure 2b - Cdc45 and Mcm7 run on same gel. Membrane cut for western blotting. Figure 2c - all samples run on same gel except Sld5. Extended Data Fig. 7g - Ctf4 and Hrt1 run on separate gels. Extended Data Fig. 7h - Mcm7 and Hrt1 run on separate gels. Extended Data Fig. 11c - Cdc45 and Mcm7 run on same gel. Membrane cut for western blotting. Extended Data Fig. 11e - all samples run on same gel. Membrane cut for western blotting. * is rabbit IgG.

Supplementary Figure 2. Gating strategy for analyses of flow cytometry data in Extended Data Fig. 7i, as exemplified for wildtype cells. **a,** An initial gate was manually applied, based on the forward (FSC-A) and side scatter (SSC-A) properties, to remove cell debris and dead cells (bottom left corner of plot). **b,** A gate was then manually applied to DNA content histograms (propidium iodide (PI-A)) to quantify the percentage of cells with 1C DNA content, representative of cells arrested in G1-phase of the cell cycle. All steps were performed using FlowJoTM v10.8 Software (BD Life Sciences).

References for Supplementary Information

1 Yeeles, J. T. P., Janska, A., Early, A. & Diffley, J. F. X. How the Eukaryotic Replisome Achieves Rapid and Efficient DNA Replication. *Mol Cell* **65**, 105-116, doi:10.1016/j.molcel.2016.11.017 (2017).

2 Yeeles, J. T., Deegan, T. D., Janska, A., Early, A. & Diffley, J. F. Regulated eukaryotic DNA replication origin firing with purified proteins. *Nature* **519**, 431-435, doi:10.1038/nature14285 (2015).

3 Taylor, M. R. G. & Yeeles, J. T. P. The Initial Response of a Eukaryotic Replisome to DNA Damage. *Mol Cell* **70**, 1067-1080 e1012, doi:10.1016/j.molcel.2018.04.022 (2018).

4 Deegan, T. D., Baxter, J., Ortiz Bazan, M. A., Yeeles, J. T. P. & Labib, K. P. M. Pif1-Family Helicases Support Fork Convergence during DNA Replication Termination in Eukaryotes. *Mol Cell*, doi:10.1016/j.molcel.2019.01.040 (2019).

5 Deegan, T. D., Mukherjee, P. P., Fujisawa, R., Polo Rivera, C. & Labib, K. CMG helicase disassembly is controlled by replication fork DNA, replisome components and a ubiquitin threshold. *eLife* **9**, e60371, doi:10.7554/eLife.60371 (2020).

6 Guilliam, T. A. & Yeeles, J. T. The eukaryotic replisome tolerates leading-strand base damage by replicase switching. *The EMBO Journal* **40**, e107037, doi:https://doi.org/10.15252/embj.2020107037 (2021).

7 Baretić, D. *et al.* Cryo-EM Structure of the Fork Protection Complex Bound to CMG at a Replication Fork. *Molecular Cell* **78**, 926-940.e913, doi:https://doi.org/10.1016/j.molcel.2020.04.012 (2020).

8 Jones, M. L., Baris, Y., Taylor, M. R. G. & Yeeles, J. T. P. Structure of a human replisome shows the organisation and interactions of a DNA replication machine. *The EMBO Journal*, e108819, doi:10.15252/embj.2021108819 (2021).

9 Zivanov, J. *et al.* New tools for automated high-resolution cryo-EM structure determination in RELION-3. *Elife* **7**, doi:10.7554/eLife.42166 (2018).

10 Zheng, S. Q. *et al.* MotionCor2: anisotropic correction of beam-induced motion for improved cryo-electron microscopy. *Nat Methods* **14**, 331-332, doi:10.1038/nmeth.4193 (2017).

11 Rohou, A. & Grigorieff, N. CTFFIND4: Fast and accurate defocus estimation from electron micrographs. *Journal of Structural Biology* **192**, 216-221, doi:https://doi.org/10.1016/j.jsb.2015.08.008 (2015).

12 Zhang, k. Gautomatch, <https://www2.mrc-lmb.cam.ac.uk/research/locally-developed-software/zhang-software/>. (2020).

13 Nakane, T., Kimanius, D., Lindahl, E. & Scheres, S. H. Characterisation of molecular motions in cryo-EM single-particle data by multi-body refinement in RELION. *Elife* **7**, doi:10.7554/eLife.36861 (2018).

14 Kucukelbir, A., Sigworth, F. J. & Tagare, H. D. Quantifying the local resolution of cryo-EM density maps. *Nat Methods* **11**, 63-65, doi:10.1038/nmeth.2727 (2014).

15 Liebschner, D. *et al.* Macromolecular structure determination using X-rays, neutrons and electrons: recent developments in Phenix. *Acta Crystallographica Section D* **75**, 861-877, doi:doi:10.1107/S2059798319011471 (2019).

16 Zhang, K. Gctf: Real-time CTF determination and correction. *J Struct Biol* **193**, 1-12, doi:10.1016/j.jsb.2015.11.003 (2016).

17 Punjani, A., Rubinstein, J. L., Fleet, D. J. & Brubaker, M. A. cryoSPARC: algorithms for rapid unsupervised cryo-EM structure determination. *Nature Methods* **14**, 290-296, doi:10.1038/nmeth.4169 (2017).

18 Emsley, P., Lohkamp, B., Scott, W. G. & Cowtan, K. Features and development of Coot. *Acta Crystallogr D Biol Crystallogr* **66**, 486-501, doi:10.1107/S0907444910007493 (2010).

19 Croll, T. I. ISOLDE: a physically realistic environment for model building into low-resolution electron-density maps. *Acta Crystallogr D Struct Biol* **74**, 519-530, doi:10.1107/S2059798318002425 (2018).

20 Afonine, P. V. *et al.* Real-space refinement in PHENIX for cryo-EM and crystallography. *Acta Crystallogr D Struct Biol* **74**, 531-544, doi:10.1107/S2059798318006551 (2018).

21 Pettersen, E. F. *et al.* UCSF Chimera--a visualization system for exploratory research and analysis. *J Comput Chem* **25**, 1605-1612, doi:10.1002/jcc.20084 (2004).

22 Zhang, Y. I-TASSER server for protein 3D structure prediction. *BMC Bioinformatics* **9**, 40, doi:10.1186/1471-2105-9-40 (2008).

23 Roy, A., Kucukural, A. & Zhang, Y. I-TASSER: a unified platform for automated protein structure and function prediction. *Nat Protoc* **5**, 725-738, doi:10.1038/nprot.2010.5 (2010).

24 Yang, J. *et al.* The I-TASSER Suite: protein structure and function prediction. *Nat Methods* **12**, 7-8, doi:10.1038/nmeth.3213 (2015).

25 Goswami, P. *et al.* Structure of DNA-CMG-Pol epsilon elucidates the roles of the non-catalytic polymerase modules in the eukaryotic replisome. *Nat Commun* **9**, 5061, doi:10.1038/s41467-018-07417-1 (2018).

26 Zheng, N. *et al.* Structure of the Cul1-Rbx1-Skp1-F boxSkp2 SCF ubiquitin ligase complex. *Nature* **416**, 703-709, doi:10.1038/416703a (2002).

27 Drozdetskiy, A., Cole, C., Procter, J. & Barton, G. J. JPred4: a protein secondary structure prediction server. *Nucleic Acids Res* **43**, W389-394, doi:10.1093/nar/gkv332 (2015).

28 Martin, E. C. *et al.* LRRpredictor-A New LRR Motif Detection Method for Irregular Motifs of Plant NLR Proteins Using an Ensemble of Classifiers. *Genes (Basel)* **11**, doi:10.3390/genes11030286 (2020).

29 Orlicky, S., Tang, X., Willems, A., Tyers, M. & Sicheri, F. Structural basis for phosphodependent substrate selection and orientation by the SCFCdc4 ubiquitin ligase. *Cell* **112**, 243-256, doi:10.1016/s0092-8674(03)00034-5 (2003).

30 Schulman, B. A. *et al.* Insights into SCF ubiquitin ligases from the structure of the Skp1-Skp2 complex. *Nature* **408**, 381-386, doi:10.1038/35042620 (2000).

31 Williams, C. J. *et al.* MolProbity: More and better reference data for improved all-atom structure validation. *Protein Sci* **27**, 293-315, doi:10.1002/pro.3330 (2018).

32 Berman, H., Henrick, K. & Nakamura, H. Announcing the worldwide Protein Data Bank. *Nat Struct Biol* **10**, 980, doi:10.1038/nsb1203-980 (2003).

33 Sorzano, C. O. *et al.* XMIPP: a new generation of an open-source image processing package for electron microscopy. *J Struct Biol* **148**, 194-204, doi:10.1016/j.jsb.2004.06.006 (2004).

34 Tang, G. *et al.* EMAN2: an extensible image processing suite for electron microscopy. *J Struct Biol* **157**, 38-46, doi:10.1016/j.jsb.2006.05.009 (2007).

35 Scheres, S. H. RELION: implementation of a Bayesian approach to cryo-EM structure determination. *J Struct Biol* **180**, 519-530, doi:10.1016/j.jsb.2012.09.006 (2012).

36 Cardote, T. A. F., Gadd, M. S. & Ciulli, A. Crystal Structure of the Cul2-Rbx1-EloBC-VHL Ubiquitin Ligase Complex. *Structure* **25**, 901-911 e903, doi:10.1016/j.str.2017.04.009 (2017).

37 Kim, Y. K. *et al.* Structural basis of intersubunit recognition in elongin BC-cullin 5-SOCS box ubiquitin-protein ligase complexes. *Acta Crystallogr D Biol Crystallogr* **69**, 1587-1597, doi:10.1107/S0907444913011220 (2013).

38 Goddard, T. D. *et al.* UCSF ChimeraX: Meeting modern challenges in visualization and analysis. *Protein Sci* **27**, 14-25, doi:10.1002/pro.3235 (2018).

39 Krissinel, E. & Henrick, K. Inference of Macromolecular Assemblies from Crystalline State. *Journal of Molecular Biology* **372**, 774-797, doi:https://doi.org/10.1016/j.jmb.2007.05.022 (2007).
