# Supplementary material for: A conserved mechanism for regulating replisome disassembly in eukaryotes
Source: Nature. 2021 Oct 26;600(7890):743–7. doi: 10.1038/s41586-021-04145-3 (PMC8695382; doi:10.1038/s41586-021-04145-3)
Supplement: Supplementary file 2 — Reporting Summary [file 41586_2021_4145_MOESM2_ESM.pdf]

nature portfolio

Corresponding author(s): Joseph T.P. Yeeles, Tom D. Deegan

Last updated by author(s): Jul 20, 2021

Reporting Summary

Nature Portfolio wishes to improve the reproducibility of the work that we publish. This form provides structure for consistency and transparency in reporting. For further information on Nature Portfolio policies, see our Editorial Policies and the Editorial Policy Checklist.

Statistics

For all statistical analyses, confirm that the following items are present in the figure legend, table legend, main text, or Methods section.

n/a	Confirmed	
☒	☐	The exact sample size (*n*) for each experimental group/condition, given as a discrete number and unit of measurement
☐	☒	A statement on whether measurements were taken from distinct samples or whether the same sample was measured repeatedly
☒	☐	The statistical test(s) used AND whether they are one- or two-sided<br>*Only common tests should be described solely by name; describe more complex techniques in the Methods section.*
☒	☐	A description of all covariates tested
☒	☐	A description of any assumptions or corrections, such as tests of normality and adjustment for multiple comparisons
☒	☐	A full description of the statistical parameters including central tendency (e.g. means) or other basic estimates (e.g. regression coefficient) AND variation (e.g. standard deviation) or associated estimates of uncertainty (e.g. confidence intervals)
☒	☐	For null hypothesis testing, the test statistic (e.g. *F*, *t*, *r*) with confidence intervals, effect sizes, degrees of freedom and *P* value noted<br>*Give P values as exact values whenever suitable.*
☒	☐	For Bayesian analysis, information on the choice of priors and Markov chain Monte Carlo settings
☒	☐	For hierarchical and complex designs, identification of the appropriate level for tests and full reporting of outcomes
☒	☐	Estimates of effect sizes (e.g. Cohen's *d*, Pearson's *r*), indicating how they were calculated

Our web collection on statistics for biologists contains articles on many of the points above.

Software and code

Policy information about availability of computer code

Data collection	EPU-2 (Thermo-Fisher Scientific)
Data analysis	RELION-3.0 and -3.1; CryoSPARC-2; Gautomatch-0.53 and -0.56; MotionCor-2; CTFFIND-4.1; Gctf-1.18; EMAN-2; Xmipp-3; ISOLDE-1.0.1; COOT-0.9.5; UCSF ChimeraX-1.0; UCSF Chimera-1.8.1; Phenix-1.19; Prism-8.0.0; FlowJo-10.8; PyMOL-2.4.1

For manuscripts utilizing custom algorithms or software that are central to the research but not yet described in published literature, software must be made available to editors and reviewers. We strongly encourage code deposition in a community repository (e.g. GitHub). See the Nature Portfolio guidelines for submitting code & software for further information.

Data

Policy information about availability of data

All manuscripts must include a data availability statement. This statement should provide the following information, where applicable:

- Accession codes, unique identifiers, or web links for publicly available datasets
- A description of any restrictions on data availability
- For clinical datasets or third party data, please ensure that the statement adheres to our policy

Cryo-EM density maps of the yeast replisome-SCFDia2 complex on dsDNA have been deposited in the Electron Microscopy Data Bank (EMDB) under the following accession numbers: EMD-13495 (full complex unsharpened map, conformation I); EMD-13496 (full complex sharpened map, conformation I); EMD-13497 (multi-body refinement [MBR], MCM N-tier, conformation I); EMD-13498 (MBR, MCM C-tier, conformation I); EMD-13500 (full complex unsharpened map, conformation II); EMD-13512 (full complex sharpened map, conformation II); EMD-13513 (MBR, MCM N-tier, conformation II); EMD-13514 (MBR, MCM C-tier, conformation II); EMD-13515 (MBR, Dia2-Skp1); EMD-13516 (MBR, Cdc45-GINS-Ctf4-Dpb2NTD); EMD-13517 (MBR, Pol enon-Cat-Mcm5WH); EMD-13518 (full complex enriched for Csm3-Tof1); composite maps produced using Phenix combine_focused_maps have been deposited under accession numbers EMD-13537 (conformation I) and

EMD-13539 (conformation II). Cryo-EM density maps of the yeast replisome-SCFDia2 complex in the absence of DNA have been deposited in the EMDB under the following accession numbers: EMD-13519 (full complex unsharpened map) and EMD-13540 (MBR). Cryo-EM density maps of the human replisome-CUL2LRR1 complex used in model building have been deposited in the EMDB under the following accession numbers: EMD-13494 (full complex, consensus refinement), EMD-13491 (MBR, AND-1/CDC45/GINS), EMD-13490 (MBR, ELONGIN-BC/LRR1/CUL2), EMD-13492 (MBR, CUL2-RBX1). An additional map of the core human replisome not engaged by CUL2LRR1 on a DNA substrate lacking a 5′-flap has been deposited under the accession number EMD-13534. Atomic coordinates have been deposited in the Protein Data Bank (PDB) with the accession numbers 7PMK for the yeast replisome-SCFDia2 complex on dsDNA (conformation I), 7PMN for the yeast replisome-SCFDia2 complex on dsDNA (conformation II), and 7PLO for the human replisome-CUL2LRR1 complex.

Field-specific reporting

Please select the one below that is the best fit for your research. If you are not sure, read the appropriate sections before making your selection.

☒ Life sciences ☐ Behavioural & social sciences ☐ Ecological, evolutionary & environmental sciences

For a reference copy of the document with all sections, see nature.com/documents/nr-reporting-summary-flat.pdf

Life sciences study design

All studies must disclose on these points even when the disclosure is negative.

Sample size	Our study does not include cohort/population based analysis or comparison and thus does not entail predetermination of sample size. Cryo-EM data were collected, as described in methods; these numbers of micrographs were sufficient to either allow model building or comparative analysis.
Data exclusions	During processing of cryo-EM data, poor quality micrographs/particles were excluded based on manual inspection and 2D/3D classification.
Replication	Cryo-EM datasets for yeast and human complexes comprised individual sample preparations and datasets. Complex formation was found to be reproducible across multiple independent sample preparations. Details of the number of experimental repeats have been acknowledged in the relevant figure legends. All attempts at data replication were successful.
Randomization	For calculation of the resolution of the cryo-EM reconstructions, Fourier shell correlations were calculated using independent halves of the complete datasets, into which the component particles were segregated randomly.
Blinding	Our analysis did not require blinding because it did not involve human subjects or live animals.

Reporting for specific materials, systems and methods

We require information from authors about some types of materials, experimental systems and methods used in many studies. Here, indicate whether each material, system or method listed is relevant to your study. If you are not sure if a list item applies to your research, read the appropriate section before selecting a response.

Materials & experimental systems

n/a	Involved in the study	
☐	☒	Antibodies
☐	☒	Eukaryotic cell lines
☒	☐	Palaeontology and archaeology
☒	☐	Animals and other organisms
☒	☐	Human research participants
☒	☐	Clinical data
☒	☐	Dual use research of concern

Methods

n/a	Involved in the study	
☒	☐	ChIP-seq
☐	☒	Flow cytometry
☒	☐	MRI-based neuroimaging

Antibodies

Antibodies used	All polyclonal primary antibodies used in this study were raised in sheep against the indicated S. cerevisiae proteins by the Labib laboratory in conjunction with MRC PPU Reagents and Services (https://mrcppureagents.dundee.ac.uk/). Mcm5 (160), Mcm6 (161), Mcm7 (19), Cdc45 (29), Psf1 (58), Sld5 (32), Ctf4 (30), Hrt1 (203).<br>Peroxidase anti-peorxidase (Sigma, P1291) conjugate to horseradish peroxidase of anti-sheep IgG from donkey (Sigma, A3415).
Validation	With the exception of anti-Hrt1 (203), the validation of all antibodies is described in Gambus et al, Nat. Cell Biol., 2006 (doi: 10.1038/ncb1382). Anti-Hrt1 (203) was validated by immunoblotting against purified SCFDia2 and yeast whole cell extracts containing wildtype Hrt1 or 6HA-Hrt1.

Eukaryotic cell lines

Policy information about cell lines

Cell line source(s)	SF9 cells were obtained from OXFORD EXPRESSION TECHNOLOGIES, LTD, Cat No. 600100. High-5 cells (bti-tn-5b1-4) were obtained from Thermo-Scientific Cat no. B85502.
Authentication	Cell lines were not authenticated.
Mycoplasma contamination	Cell lines tested negative for mycoplasma contamination.
Commonly misidentified lines (See ICLAC register)	None

Flow Cytometry

Plots

Confirm that:

☒ The axis labels state the marker and fluorochrome used (e.g. CD4-FITC).

☒ The axis scales are clearly visible. Include numbers along axes only for bottom left plot of group (a 'group' is an analysis of identical markers).

☒ All plots are contour plots with outliers or pseudocolor plots.

☒ A numerical value for number of cells or percentage (with statistics) is provided.

Methodology

Sample preparation	For each sample, 10 million yeast cells were harvested and fixed by resuspension in 1 ml of 70% ethanol. Subsequently, 3 ml of 50 mM sodium acetate and 50mg of RNase A was added to 150 uL of fixed cells, followed by incubation at 37°C for 2 h. The cells were then pelleted and proteins degraded by incubation at 37°C for 30 min in 500 uL of 50 mM HCl containing 2.5 mg of Pepsin. Finally, cells were pelleted and then re-suspended in 1 ml of 50 mM sodium citrate containing 2 mg of propidium iodide. Samples were sonicated and then analysed.
Instrument	FACSCanto II flow cytometer (Becton Dickinson)
Software	FlowJoTM v10.8 software Software (TreeStar Inc.)
Cell population abundance	The percentage of cells in G1-phase is provided in EDF 7i.
Gating strategy	Gating was performed to remove cell debris and dead cells based on forwards and side scatter properties, as exemplified in Supplementary Fig. 2

☒ Tick this box to confirm that a figure exemplifying the gating strategy is provided in the Supplementary Information.
